# Rational Design of Weakly‐Solvating Molecules for Salt‐In‐Pre‐Ionic‐Liquid Electrolytes for Li Metal Batteries

**DOI:** 10.1002/advs.75550

**Published:** 2026-05-07

**Authors:** Bishnu P. Thapaliya, Vaidyanathan Sethuraman, Naresh C. Osti, Arvind Ganesan, K. Shawn Reeves, Michael J. Zachman, Albina Y. Borisevich, Harry M. Meyer, Xiao‐Guang Sun, Eugene Mamontov, Lei Cheng, Sheng Dai

**Affiliations:** ^1^ Chemical Sciences Division Oak Ridge National Laboratory Oak Ridge Tennessee USA; ^2^ Neutron Scattering Division Oak Ridge National Laboratory Oak Ridge Tennessee USA; ^3^ Center for Nanophase Materials Sciences Oak Ridge National Laboratory Oak Ridge Tennessee USA

**Keywords:** anodeless Li metal battery (ALMB), Li metal batteries (LMBs), molecular engineering, salt‐in‐pre‐ionic‐liquid (SIPIL), trifluoromethanesulfonamide pyrrolidine (TFMSPyr), weakly‐solvating solvent

## Abstract

Lithium metal batteries (LMBs) promise step‐changes in energy densities but suffer from poor cycle life due to unstable electrolyte‐lithium interfaces. Conventional carbonate electrolytes exhibit excessive lithium‐ion solvation and low oxidative stability, leading to rapid capacity loss. Herein, we report a rationally designed weakly‐solvating cyclic sulfonamide, 1‐trifluoromethanesulfonyl)amide pyrrolidine (TFMSPyr), which integrates an electron‐withdrawing trifluoromethanesulfonyl functional group at pyrrolidinic‐N. TFMSPyr acts as a pre‐ionic‐liquid solvent that forms intrinsically localized, anion‐dominated solvation, coupling molecular architecture, solvation topology, and transport dynamics. As a result, LiFSI based salt‐in‐pre‐ionic‐liquid (SIPIL) electrolytes exhibit high lithium‐ion transference number, oxidative stability > 5 V versus Li/Li^+^ and anion‐derived solid electrolyte interphases (SEI). Li||Cu cells with SIPIL deliver a first cycle Coulombic efficiency (CE) of ≈ 99% with average CE of 99.2% for 100 cycles, and lithium half‐cells with lithium iron phosphate (LFP) cathode exhibit 82% capacity retention after 400 cycles with CE of 99.98%. In anode‐free full cells, 95% of initial capacity is retained after 63 cycles with an average CE of 99.5%. These results demonstrate that molecular engineering of solvents offers a powerful pathway to stabilize lithium metal interfaces and enable practical Anodeless LMBs.

## Graphical abstract

The weakly‐solvating TFMSPyr electrolyte tailors the Li^+^ solvation structure by suppressing solvent coordination and promoting anion‐dominated solvation. This unique solvation environment induces preferential anion decomposition at electrode interfaces, forming robust inorganic rich S/CEI. Consequently, dendrite free lithium deposition, enhanced interfacial stability, and long cycling performance are achieved.

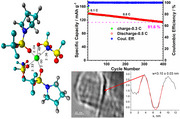

## 1 Introduction

The resurgence of interest in lithium metal batteries (LMBs), including anode‐free configurations, stems from the promise of achieving energy densities far beyond those of conventional lithium‐ion batteries (LIBs) [1, 2, 3]. However, the instability of traditional carbonate‐based LIB electrolytes against reactive lithium metal surfaces remains a critical barrier. These electrolytes exhibit high reactivity toward lithium, increased dendritic growth, continuous parasitic reactions, and unstable solid electrolyte interphase (SEI) layers, all culminating in rapid capacity loss, low Coulombic efficiency (CE), and poor cycle life [4, 5, 6, 7, 8]. Extensive efforts have been focused on electrolyte design to mitigate the above challenges.

The realization that the interfacial chemistry and electrolyte stability are governed by the solvation structures marks a paradigm shift in electrolyte design, focusing on tuning the solvation structure from solvent separated to anion dominated contact ion pairs and aggregates [9]. Therefore, researchers are focused on electrolyte formulation for enhancing the lithium metal compatibility, oxidative stability, anion rich stable SEI formation, and modification of solvent dynamics [10, 11, 12]. The electrolyte formulations mainly focused on achieving these features, including high‐concentration electrolytes (HCEs) [13, 14, 15, 16], localized high‐concentration electrolytes (LHCEs) [17, 18, 19], ionic liquid electrolytes and locally concentrated ionic electrolytes [20, 21], high entropy electrolytes [22], and fluorinated and ether based solvent systems [12, 23, 24]. While these strategies have improved lithium metal compatibility, they often suffer from drawbacks such as increased viscosity, reduced ionic conductivity, limited transference numbers, complex synthesis of fluorinated co‐solvents, and high cost, which hamper their practical deployment in full cells [25]. Recently, alternative electrolyte design concept, called weakly‐solvating electrolytes (WSEs) with low donor numbers and reduced solvent‐cation interactions has emerged, which alters the primary solvation with anion‐dominated structure without increasing the salt concentration as HCEs or LCEs [26, 27, 28]. The WSEs shown tremendous promise towards Li/Na metal batteries, under high voltage operation, low temperature [29, 30, 31, 32]. Different approaches have been investigated to formulate WSEs such as single solvent and adding co‐solvent to regulate the solvation structure. Most of the weakly solvating solvents (WSSs) and co‐solvents used to formulate WSEs are linear molecules with conformational flexibility, which makes the solvation structure less controlled [33, 34]. Having conformationally rigid single solvent WSE would be beneficial for regulating solvation structures and facilitate faster ion transport via close‐range hoping between adjacent coordination‐site to achieve excellent battery performance.

Herein, we report on the molecular engineering of conformationally rigid weakly‐solvating cyclic sulfonamide solvent, TFMSPyr, tailored to address the intrinsic deficiencies of conventional electrolytes and WSEs. The molecular architecture of TFMSPyr integrates a cyclic scaffold with electron‐withdrawing trifluoromethanesulfonyl groups, effectively suppressing its lithium‐ion solvating power. Inspired by the unique electrostatic environment of ionic liquids, we introduce a concept of pre‐ionic liquid (PIL), a class of neutral molecular solvents containing electron‐donating and electron‐withdrawing groups within the same molecular framework. The coexistence of these opposing electronic functionalities induces significant intramolecular charge separation, generating localized partial charges that mimic the electrostatic interactions present in ionic liquids. Despite being molecularly neutral, PIL creates a highly polarized solvation environment, thereby reproducing key advantages of ionic liquids while maintaining the molecular mobility of conventional molecular solvents. Besides, PIL has several advantages over ionic liquids such as lower viscosity, enhanced salt solubility and weak solvent coordination caused by dipolar solvation environment, and high Li^+^ transference number. When LiFSI salt is dissolved in TFMSPyr solvent, the resulting electrolyte is named as salt‐in‐pre‐ionic‐liquid (SIPIL) that forms intrinsically localized, anion‐dominated solvation structure, coupling molecular architecture, solvation structure, and dynamics, which enables the formation of a robust, anion‐rich S/CEI and promotes high lithium plating/stripping efficiency. The resulting WSEs demonstrates a high lithium‐ion transference number (0.6), superior oxidative stability exceeding 5 V versus Li/Li^+^, and exceptional electrochemical performance. In symmetric Li||Cu cells, the TFMSPyr‐based SIPIL electrolyte delivers an outstanding first‐cycle CE of ≈ 99%, evaluated by the stringent Aurbach method [35], and maintains an average CE of 99.2% over 100 cycles. Near‐ideal CE of 99.98% are achieved in lithium half‐cells with lithium iron phosphate (LFP) cathode, with 82% capacity retention after 400 cycles. Furthermore, in anode‐free LFP full cells, the SIPIL electrolyte enables 95% capacity retention after 63 cycles with a CE of 99.5%, highlighting its potential to enable stable and high‐energy‐density lithium metal batteries. These results demonstrate that the molecularly engineered weakly‐solvating TFMSPyr electrolytes offers a powerful pathway to overcome the interfacial instability challenges of lithium metal batteries, marking a critical step toward their practical realization.

## 2 Results and Discussion

### 2.1 Design of Weakly‐Solvating Cyclic Sulfonamide for Salt‐In‐Pre‐Ionic‐Liquid (SIPIL)

**FIGURE 1:**
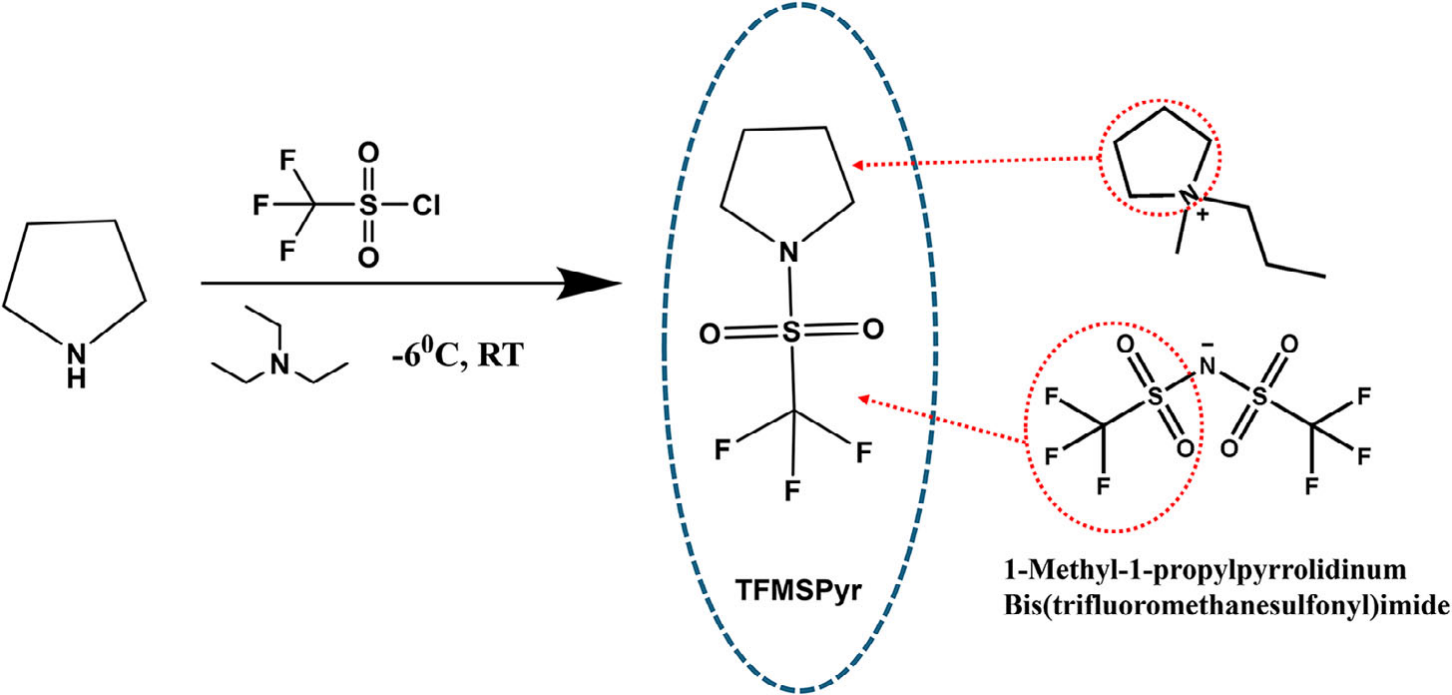
Molecular design of ionic liquid precursor (pre‐ionic‐liquid) as a solvent for the preparation of salt‐in‐pre‐ionic‐liquid (SIPIL) electrolytes for next generation lithium metal batteries.

**FIGURE 2:**
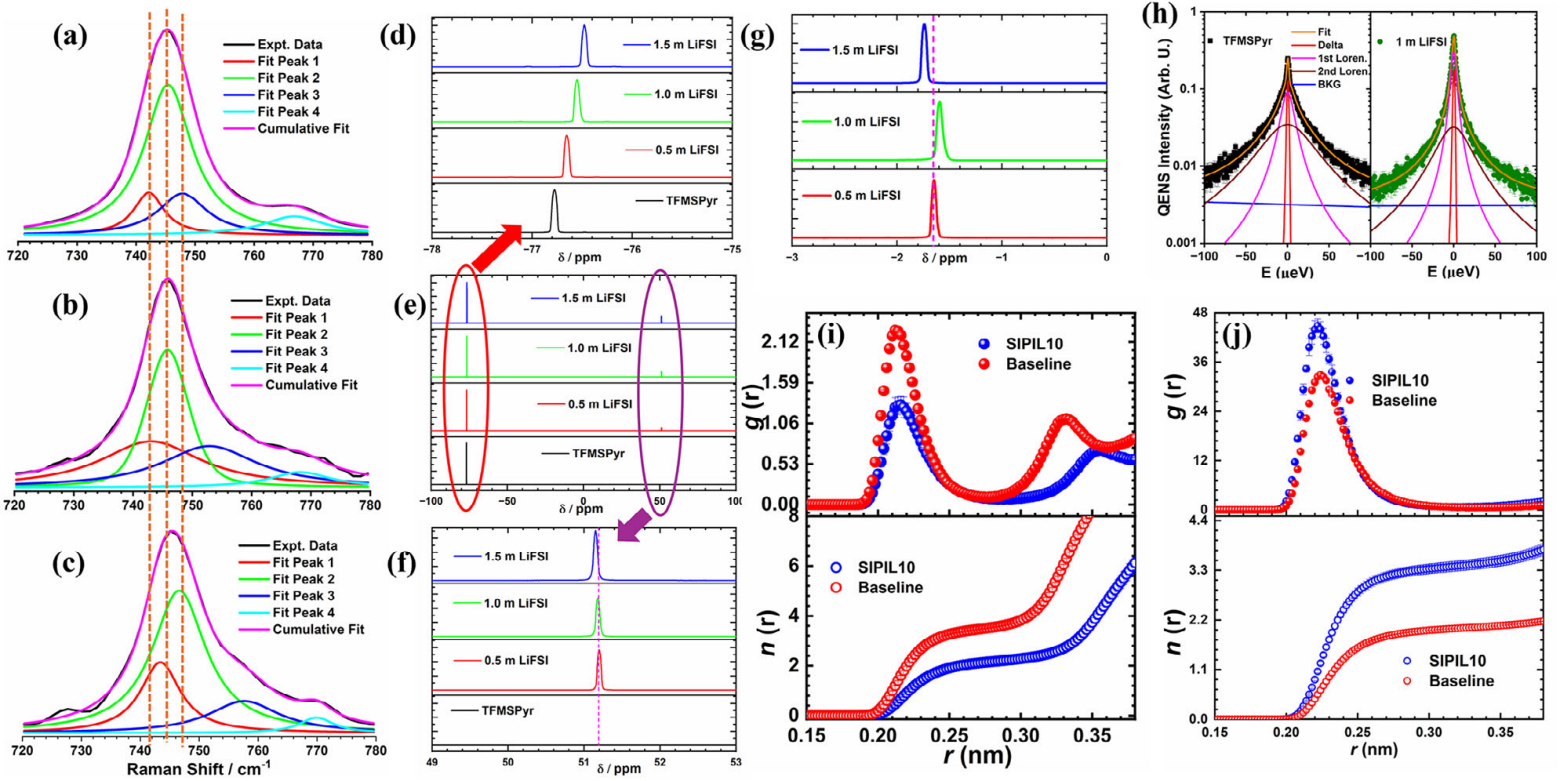
Raman spectra of (a) 0.5 m, (b) 1 m, (c) 1.5 m; (d–f) ^19^ F NMR of TFMSPyr‐and different concentration of LiFSI in TFMSPyr (d – extended region of ^19^ F of TFMSPyr and f ‒ extended region of ^19^ F NMR of FSI anion from LiFSI, (g) ^7^ Li NMR of LiFSI, (h) QENS spectra of neat TFMSPyr (left) and 1 m LiFSI in TFMSPyr (SIPIL10, right) at Q = 0.7 Å^−1^ (symbols represent the experimental data and the solid lines are the models), (i) Comparison of Radial distribution function (RDF), *g*(*r*) (top) and *n*(*r*), (bottom) of Li‐solvent, and (j) Comparison of RDF, *g*(*r*) (top) and *n*(*r*) (bottom) of Li‐anion system for TFMSPyr and EC/EMC system obtained from MD simulation.

The design of PIL aims to mimic the electrostatic solvation capability of the ionic liquids while avoiding the strong ion‐ion correlations that lead to high viscosity and limited mass transport. By embedding electron‐donating and electron‐withdrawing groups within a single molecule, PIL generates internal polarization that provides ionic liquid like electrostatic environment for dissolved salts. This molecular design enables efficient salt dissociation and weakly coordinating solvation while preserving low viscosity, enhanced ionic mobility, and transference number. Inspiring from the excellent physio‐chemical and electrochemical properties of ionic liquids for the next generation lithium metal batteries [20, 36], weakly‐solvating novel cyclic sulfonamide solvent is designed mimicking properties of 1‐methyl‐1‐propylpyrrolidinum bis (trifluoromethanesulfonyl)imide ionic liquid to overcome the low ionic conductivity, low lithium‐ion transference number [37], and low lithium plating/stripping efficiency in Li||Cu cells. Especially, alkylpyrrolidine structural moiety was chosen to synthesize weakly‐solvating solvent because of excellent electrochemical stability and lithium metal metal compability. Weakly‐solvating 1‐trifluoromethanesulfonylpyrrolidine, hereafter called TFMSPyr was designed by integrating the trifluoromethanesulfonyl (TFMS) segment with pyrrolidine architecture through the N‐atom. The design scheme and synthesis route are displayed in Figure 1. The molecular structure of TFMSPyr was determined by ^1^H, ^13^C and ^19^F NMR spectroscopy (Figure 2d–f; Figure S1).

TFMSPyr platform provides unique weak lithium solvation environments because of intramolecular charge transfer (ICT, donor‐acceptor) character of TFMSPyr, resulting from coupled electron‐donating pyrrolidinic N and electron withdrawing CF_3_SO_2_‐ group. Such ICT often produces zwitterionic‐like resonance and conformationally rigid molecular structure, resulting in lower lithium solvation abilities of N and O atoms, lower volatility, resembling the characteristics of the ionic liquid. Therefore, the solution of LiFSI salt in TFMSPyr, a fluorinated molecular ionic liquid precursor solvent, is referred as “SIPIL” (salt‐in‐pre‐ionic‐liquid) electrolytes. These SIPIL electrolytes with mixture of anionic FSI and polarized TFMS moieties have similar ion speciation in solvent‐in salt electrolytes and salts in ionic liquids electrolytes, but with significantly lower viscosity (see the Table S1) compared with its ionic liquid counterparts and higher ionic conductivities and much higher lithium‐ion transference number. Because of the combination of electrochemically stable TFMS moiety and inorganic rich SEI former FSI anion, SIPIL electrolytes results in robust S/CEI, wide electrochemical potential window, high lithium‐ion transference number, high lithium plating/stripping efficiency.

### 2.2 Molecular Level Understanding of Li^+^ Solvation and Ion Transport in SIPIL Electrolytes

A comprehensive understanding of Li^+^ solvation and transport in the TFMSPyr‐based electrolyte was achieved through a synergistic integration of Raman spectroscopy (Figure 2a–c; Figure S2), ^7^Li NMR spectroscopy (Figure 2g), ^19^F NMR spectroscopy (Figure 2d–f), Quasi‐elastic neutron scattering (QENS) experiment (Figure 2h), and molecular dynamics (MD) simulations (Figure 2i,j), enabling direct correlation between local coordination chemistry, mesoscale dynamics, and macroscopic transport behavior. Raman spectroscopy first establishes the primary solvation structure, revealing that the S‐N‐S vibrational band of the FSI anion shifts from ≈ 774 cm^−1^ (LiFSI salt, Figure S3b) to ≈ 742 – 757 cm^−1^ (Figure 2a–c) upon dissolution in TFMSPyr, whose S‐N‐S vibration band lies in the same region(Figure S3a), indicating dominant Li^+^‐FSI^−^ interaction. As shown in Figure 2a–c, deconvolution of the 720 – 780 cm^−1^ regions confirms that the solvation environment is overwhelmingly composed of of only CIPs (red curve, 742–743 cm^−1^), AGGs (blue curve, 747 –757 cm^−1^) [38], and green curve at ≈ 745 cm^−1^ could be the mixture of the S‐N‐S bending vibration for CF_3_SO_2_N group of TFMSPyr solvent and AGGs, indicating weakly solvating power of the TFMSPyr solvent (lower donor number of N & O because of electron withdrawing CF_3_SO_2_‐ group). No discernible bands were observed in the lower‐wavenumber region corresponding to free anions or SSIPs, suggesting that Li^+^ exists predominately within multi‐anion coordination domains. Importantly, the invariance in peak position across concentrations (see fitted spectral pattern in Table S2) suggests that the Li^+^ coordination motif remains structurally conserved, with increasing salt concentration primarily enhancing network connectivity rather than altering local coordination geometry. This anion‐dominated coordination environment identified by Raman spectroscopy is directly corroborated by multinuclear NMR Spectroscopy. The progressive line broadening and upfield shift in ^7^Li NMR spectra (Figure 2g) indicate reduced Li^+^ ion exchange dynamics between coordination sites and enhanced Li^+^‐FSI interaction and weaker solvation (Li^+^‐TFMSPyr interactions), consistent with confinement within multi‐anion coordination domains. Complementarily, ^19^F NMR shows opposite chemical shift trends for TFMSPyr (slightly downfield shift from pure solvent to LiFSI solution) and FSI^−^ species (slightly upfield shift Figure 2d–f), confirming that FSI‐anions increasingly participate in the primary solvation shell while solvent molecules remain weakly coordinated. Together, Raman and NMR provide a consistent picture of a weakly solvating solvent environment that enforces Li^+^ ‐FSI^−^ dominated coordination. This multi anion‐bridged solvation structure is crucial for the formation of robust S/CEI, enhance the reductive and oxidative stability of the electrolyte, lithium‐ion transference number caused by lowered desolvation barriers of Li ion.

The dynamic consequences of this structural motif are captured by QENS and PFG‐NMR measurements. QENS spectra (Figure 2h, left for neat solvent and Figure 2h, right for solution, along with the model fit) exhibit significant narrowing upon salt addition, with reduced half‐width at half‐maximum (HWHM) values (Figure S4a) indicating suppressed translational mobility of solvent molecules. The necessity of two Lorentzian components to fit the QENS data, as is typical for many ionic liquids and salt systems [39, 40] further reveals coexisting dynamic regimes: (i) slow translational diffusion associated with extended ion‐coordinated domains and (ii) fast localized rattling motions of the solvent molecules within transient cages formed by neighboring molecules [41, 42]. Furthermore, the Q‐dependence of the HWHMs for the fast process (see Figure S4b) does not decay to zero in the limit of Q = 0. This suggests that the fast process is, indeed, localized in character, which can be attributed to the in‐transient‐cage rattling motion of the solvent molecules [40, 41, 42]. On the other hand, the HWHM of the slow process (Figure S4a) exhibits a strong dependence on Q^2^ at low Q. Based on these results, QENS spectra further confirmed the formation of aggregated domains, as evidenced by reduced translational diffusion coefficients relative to the neat solvent. The HWHM of the quasielastic component decreased systematically from neat TFMSPyr to 1.0 m LiFSI, confirming stronger Li^+^‐anion correlations and suppressed translational mobility. Similar to previous studies [43, 44], the self‐diffusion coefficients of the solvent molecules decreased by a factor of 2 from neat TFMSPyr to the 1.0 m LiFSI solution (Figure S4a), corroborating the restricted motion within extended anion‐bridged clusters. These findings align quantitatively with pulse field gradient (PFG)‐NMR results which show decreased solvent diffusion coefficients with increasing salt concentration, indicating increased ion‐ion interaction that results in higher Li‐ion transference number (Table S3) compared to other dual salt solutions reported in literature [37, 45, 46]. Thus, anion‐bridged solvation network identified by Raman/NMR manifests as spatially confined, dynamically heterogeneous domains, directly impacting ion transport.

The Raman, NMR, QENS backed Li^+^‐anion dominated coordination environment in primary solvation shells was further validated by molecular dynamics (MD) simulation. MD simulation results reveal distinct Li^+^‐solvation behaviors in LiFSI depending on solvent chemistry. The radial distribution function of TFMSPyr shows a lower intensity first peak with *g*(*r*) ≈ 1.4 (cf. Figure 2i, top panel) compared with EC/EMC solvents (g(r)∼2.2), yielding a significantly weaker solvent coordinated solvation structure. The coordination number (CN) corresponds to the value of *n*(*r*) at *r*  =  0.27 nm, the first minimum in *g*(*r*). The CN for TFMSPyr ≈ 2.4 (cf. Figure 2i, bottom panel) is lower compared with that of the mixture of ethylene carbonate and ethyl methyl carbonate (CN ≈ 3.55), which exhibit strongly solvent dominated compact cation solvation structure. In addition, RDF of Li^+^‐O (FSI) in TFMSPyr shows much higher and sharper first shell peak (Figure 2j, top panel), indicating a strong, FSI dominated environment (CN_FSI_ ≈ 3.1, Figure 2j, bottom panel) compared to carbonate counterpart (CN_FSI_ ≈ 1.74). MD simulation indicates that TFMSPyr produces extended Li‐FSI interacting network that arises from the lower coordinating strength of TFMSPyr. Solvation pattern of TFMSPyr shifted towards anion dominated primary solvation with [Li(FSI)_∼2.5_]^1.5−^‐type aggregates, consistent with experimentally observed CIP/AGGs dominance and reduced solvent participation. Importantly, MD‐derived structural descriptors directly rationalize the experimental transport behavior: the extended Li‐FSI network explains the reduced solvent mobility observed in QENS and PFG‐NMR, while the lower solvent coordination facilitates reduced desolvation barriers, favors anion derived electrode‐electrolyte interface (further validated by DFT calculation described later), and enhanced Li‐ion transference number. This establishes a direct structure‐dynamics‐transport relationship across techniques.

Collectively, Raman, NMR, QENS, and MD simulation converge on a unified solvation model in which Li^+^ is embedded within percolating, anion‐bridged coordination networks characterized by persistent CIPs/AGGs as described by a [Li(FSI)_∼2.5_]^1.5−^ structural motif (Figure S5). The absence of free anions/SSIP species across concentrations suggests that further salt addition primarily increases the connectivity of preexisting CIP/AGGs rather than altering the primary coordination geometry. This behavior closely resembles localized high‐concentration electrolytes (LHCEs)—yet emerges here without diluent engineering, highlighting the role of solvent molecular design—in which isolated anion‐rich clusters dominate solvation and transport [47]. From a broader perspective, this integrated analysis demonstrates that molecular‐level solvation topology dictates mesoscale dynamics and ultimately governs macroscopic transport properties, providing a coherent framework for designing weakly solvating electrolytes that simultaneously achieve high stability, high Li^+^ transference number, and favorable interphase chemistry. This work bridges the gap between localized high‐concentration and ionic‐liquid electrolyte chemistries, offering a blueprint for developing advanced energy materials that unite high stability, safety, and cation‐selective transport for future lithium‐metal and high‐voltage batteries.

### 2.3 Physicochemical and Electrochemical Properties of SIPIL

**FIGURE 3:**
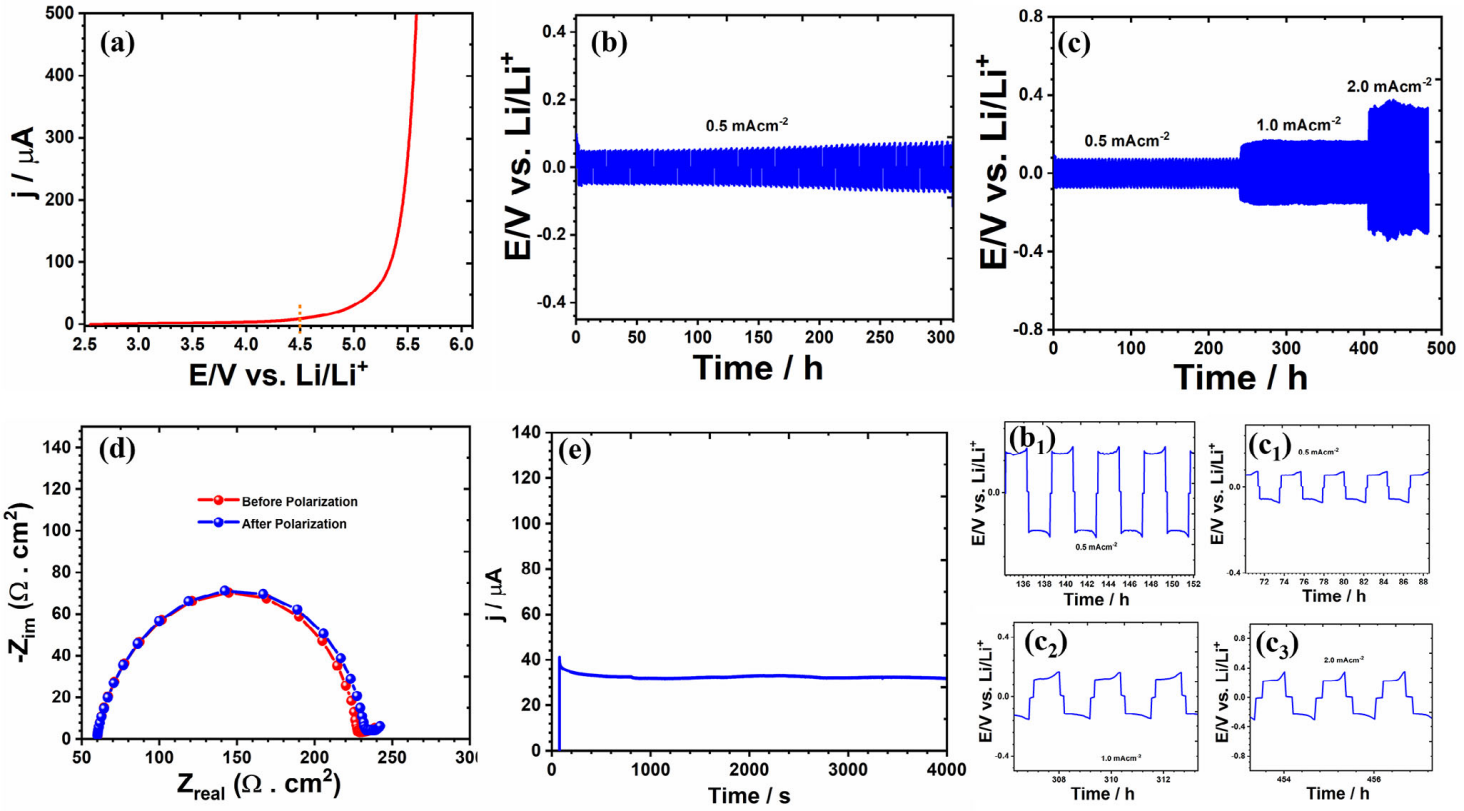
(a) Linear sweep voltammetry (LSV) plot of Li||SIPIL10||SS (stainless steel) cell with 10.0 mV s^−1^ applied potential at room temperature, showing stable current until ≤ 5.0 V and exponential increase of the current beyond 5.0 V suggesting SIPIL's high oxidation stability. Lithium metal plating / stripping performance of SIPIL10 in Li||SIPIL10||Li symmetrical cell (b) at scan rate of 0.5 mA.cm^−2^ and (b_1_) extended voltage profile from Figure 2b, (c) at different scan rates (0.5, 1.0, and 2.0 mA cm^−2^) with plating/stripping of 1.0 mAh cm^−2^ of Li at room temperature and (c_1_, c_2_, and c_3_) extended voltage profile at different current densities from Figure 2c, (d) Alternating current electrochemical impedance spectra of Li||SIPIL10||Li before and after direct current polarization (e) applying a bias potential of 10 mV.

SIPIL10 exhibit good Li^+^ conductivity of 0.86 mS cm^−1^ at 27 °C and 1.37 mS cm^−1^ at 57 °C (Figure S6a and Table S4). The SIPIL electrolytes also show good oxidation stability (> 5.0 V, Figure 3a) and resistance to solvent reduction at lithium metal interface as indicated by cyclic voltammetry of SIPIL between Li||SS electrodes (Figure S6b). The wider electrochemical stability window couldn't be only described by HOMO (−7.686 eV) ‐LUMO (−0.28 eV) energy gap of TFMSPyr (Figure S7a, b), which is narrower than traditional carbonates solvents [48, 49]. The narrower HOMO‐LUMO energy gap could be because of intramolecular charge‐transfer (ICT) [50, 51], between pyrrolidinic‐N (donor) and CF_3_SO_2_‐(acceptor) group, resulting in raising HOMO and lowering LUMO. However, actual HOMO‐LUMO gap could be altered by the formation of extended Li‐FSI AGGs solvation structure which could lower the HOMO and increase the LUMO, resulting in wider electrochemical potential window. The Li transference number was calculated by Bruce‐Vincent method (Figure 3d,e) and was found to be 0.61 at room temperature, which is slightly higher than the transference number calculated by PFG NMR method for SIPIL10 (Table S3). Similar trend was reported for other electrolytes in the literature [52]. Such a high transference number facilitates the uniform current distribution across the electrode area suppressing polarization and dendrite formation. The anion‐dominated CIP/AGGs in primary solvation will lower the desolvation barriers, leading to anion immobilization while maintaining dynamic Li^+^ exchange between adjacent coordination sites, thereby accounting for the high Li^+^ transference numbers. The lithium metal compatibilities of SIPIL were further investigated by cycling symmetric Li||Li coin cells at different current densities. The voltage versus time plot shows a stable voltage response when Li||Li symmetric cell was cycled at 0.5 mA cm^−2^ current density by plating/stripping 1.0 mAh cm^−2^ lithium for > 300 h without voltage spike (Figure 3b, b_1_(expanded E/V vs. time from Figure 3b)). Similarly, Li||Li symmetric cell exhibited stable voltage profile when the coin cell was cycled at different current densities (0.5, 1.0, 2.0 mA cm^−2^). Voltage response increases with increasing current densities which was expected as the rate of charge transferred was kinetically limited because of the low ionic conductivities (Figure 3c, c1–c3) (expanded E/V vs time plot from Figure 3c)). Voltage polarization remains stable up to 2.0 mA cm^−2^ and a large increase in voltage and high fluctuation observed when current density changed to 5 mA cm^−2^, suggesting limiting current density has reached. Therefore, the limiting current density was empirically estimated to be ≤ 5 mA cm^−2^. This high limiting current density of SIPIL electrolytes even with low ionic conductivity is due to its high reductive stability and lithium‐ion transference number caused by anion‐dominated solvation structure that resulted in robust anion derived SEI on Li metal that suppresses the dendrite growth.

**FIGURE 4:**
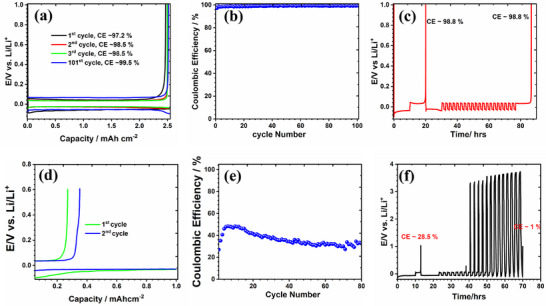
Electrochemical performance of lithium metal plating / stripping on a copper in a two electrode Li|Cu, where Li as counter and reference and Cu as a working electrode. (a) Li plating / stripping voltage profiles, (b) coulombic efficiency as a function of cycle number, and (c) lithium plating / stripping efficiency measured by Aurbach methods for Li||SIPIL10||Cu cell, (d) Li plating / stripping voltage profiles, (e) coulombic efficiency as a function of cycle number, and (f) lithium plating / stripping efficiency measured by Aurbach methods for Li||baseline electrolyte||Cu cell.

A good electrolyte should form robust SEI at the Li electrode surface to limit or prevent the reactivity at the Li‐electrolyte interface that ensures the suppression or prevention of the Li metal dendrite growth, high coulombic efficiency, and longevity of the LMB's life. The stability of the lithium metal anode and cycling efficiency is usually measured by plating and stripping fixed amount of Li on the Cu electrode in a Li||Cu cell. The Li plating/stripping coulombic efficiency and cycling stability of SIPIL10 was measured in Li||Cu coin cell configuration by plating/stripping 2.5 mAh cm^−2^ Li at a current density of 0.5 mA cm^−2^. SIPIL10 exhibits first cycle CE of 97.2% with polarization potential ≈ 90 mV. It is worth noting that the Cu electrode (thickness ≈ 20 µm) used in this experiment was used as received without any surface treatment. The first cycling Li loss and slightly higher polarization potential could be due to the oxide layer present on the Cu surface. The CE in the subsequent cycle reaches to 98.5% with overpotential < 60 mV and average CE of 99.5% was obtained after 101 cycles with overpotential of only 120 mV (Figure 4a,b, whereas 1.2 M LiPF_6_ in EC/EMC (ethylene carbonate/ethylmethyl carbonate ‐3:7 by volume) exhibits first cycle CE < 30% and never exceeds 50% CE throughout the testing period (Figure 4d,e). In conventional Li||Cu plating/stripping tests, the measured CE is strongly influenced by reactions on the fresh Cu surface, such as SEI formation, which can underestimate the true reversibility of the lithium deposition. In modified Aurbach method, a large amount of lithium (5 mAh cm^−2^) first plated and stripped to stabilize the Cu surface, after which lithium is replated (5 mAh cm^−2^) and repeatedly stripped in small fractions (0.5 mAh cm^−2^) while keeping a lithium reservoir on the substrate. This approach minimizes the Cu surface effect and enables more reliable calculation of the average lithium plating/stripping CE by quantifying the cumulative irreversible lithium loss over multiple cycles (20 cycles in present test). Almost 99.0% initial CE was delivered by SIPIL10 when measured by modified Aurbach method [35] to negate the effect of Cu electrode surface (Figure 4c), whereas 1.2 M LiPF_6_ electrolyte isn't stable enough during testing period due to excessive interfacial parasitic reaction (Figure 4f). CE and lithium metal stability of SIPIL10 is superior to highly concentrated electrolyte (4 M LiFSI/DME), (initial CE of 77.9% with ≈ 80 mV overpotential when plating and stripping 2.5 mAh cm^−2^ Li and CE of 94% by Aurbach testing method, Figure S8). Even lower concentration SIPIL (SIPIL5, 0.5 m LiFSI‐TFMSPyr) exhibits much higher CE (first cycle CE of 94%, ≈ 98% CE by Aurbach method, and Av. CE > 99.0% after 80^th^ cycle) and lithium metal stability compared to 4 M LiFSI/DME (Figure S9). The reduced availability of TFMSPyr molecules in primary solvation shells and the conformational rigidity of the cyclic sulfonamide structure further stabilize the solvation environment, enabling uniform Li plating/stripping during galvanostatic cycling.

**FIGURE 5:**
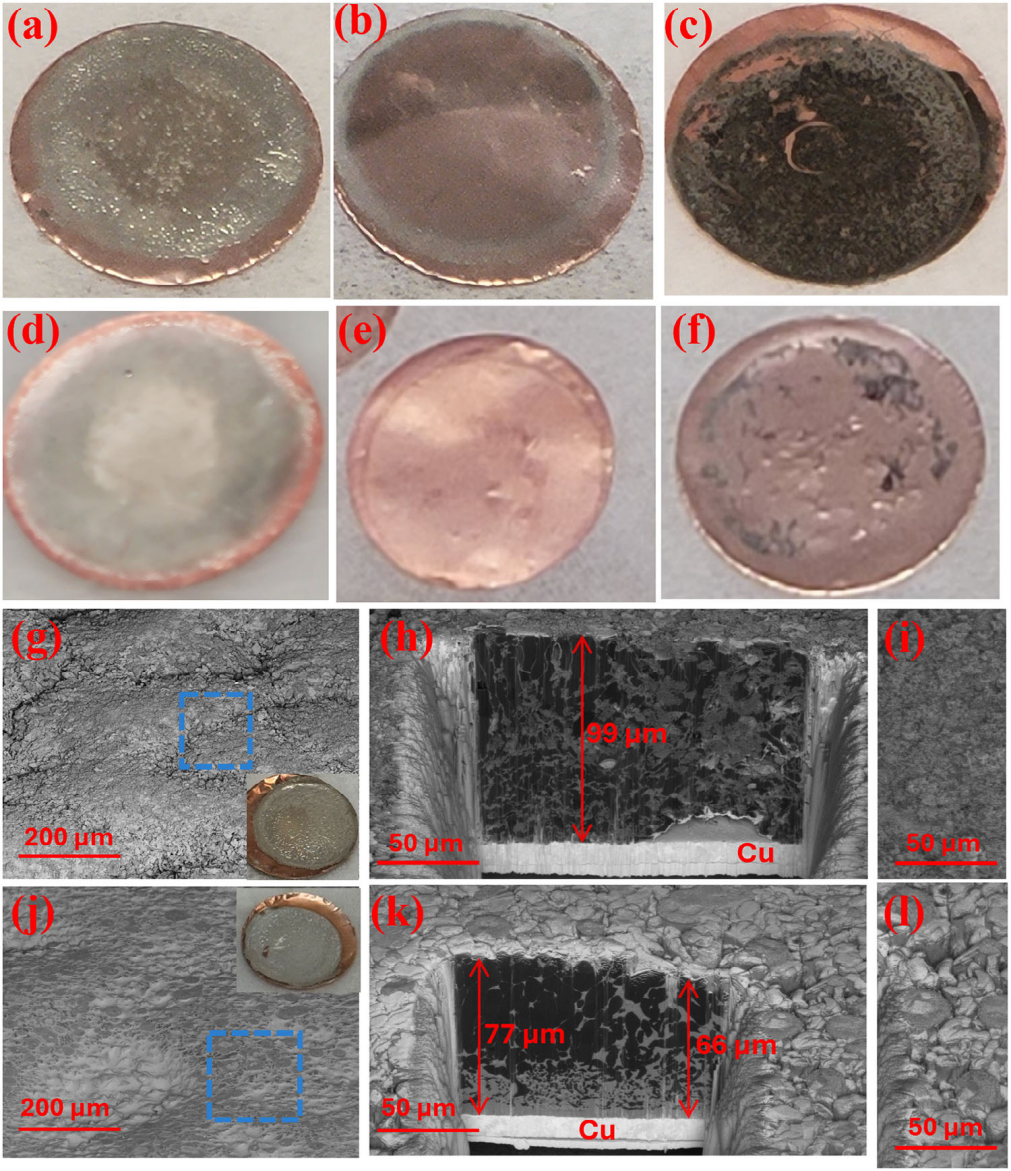
Optical images of the Cu electrodes from Li||Cu cell cycled at 0.5 mA cm^−2^ plating 1 mAh cm^−2^ Li after: (a) first plating, (b) first stripping, and (c) 50^th^ stripping cycle with baseline electrolyte; (d) first plating, (e) first stripping, and (f) 50^th^ stripping cycle with SIPIL10 electrolyte. Cryo‐PFIB/SEM BSE of Cu electrodes from Li||Cu cell after plating 10.0 mAh cm^−2^ Li cycled at 0.5 mA cm^−2^: (g) top and (h) cross‐sectional views of deposited Li in the baseline electrolyte. (i) top and (j) cross‐sectional views of deposited Li in SIPIL10.

The evolution of anode morphologies of the Anodeless cells after Li plating and stripping on the Cu substrate were further investigated to obtain a better understanding of the effects of the SIPIL on passivating the interface reactivity, stabilizing the SEI for longevity of the LMB performance. Figure 5a–c represents the digital photos of the Cu substrate after first lithium plating, first stripping and 50^th^ cycle after stripping, respectively, with 1.2 M LiPF_6_ in EC/EMC, whereas Figure 5d‐f shows the digital photos of the Cu substrate with respective cycles with SIPIL10. Remarkable differences in morphologies of Li deposits between SIPIL10 and 1.2 M LiPF_6_ in EC/EMC electrolytes were notices both by digital images and scanning electron microscopy images (Figure 5g–j). The thick accumulation of the SEI layer on the copper electrode after 50^th^ stripping cycle (Figure 5c) for the baseline electrolyte indicates severe side reactions of solvent decompositions at the interface, causing high irreversible lithium stripping. However, SIPIL10 exhibits minimal SEI build up (Figure 5f) on the copper electrode even after 50^th^ stripping cycle that is due to slow degradation of the FSI anion rather than the TFMSPyr solvent, indicating that excellent lithium plating/stripping efficiency links to excellent redox stability of TFMSPyr and compatibility with Li metal. For the morphology analysis, 10 mAh cm^−2^ Li was deposited on the Cu substrate at a current density of 0.5 mA cm^−2^ for 20 hrs (Figure 5g, i with respective optical images of the Cu electrodes (with plated lithium showing in the inset). Cryogenic plasma focused ion beam/scanning electron microscopy (cryo‐PFIB/SEM) allowed cross‐sections to be produced entirely through the lithium on copper electrodes and imaged at high resolution. The blue boxes in Figure 5g,i indicate the trenched area shown in Figure 5h,j. In both Li‐plating scenarios (1 or 10 mAh cm^−2^), porous/loose deposit structures with dendritic Li were observed in the baseline electrolyte (Figure 5g,h), whereas compact aggregates of large nodule‐like appearance were detected in SIPIL10 (Figure 5i,j). The thickness of deposited lithium with baseline electrolyte is ≈99 µm (Figure 5h), approximately twice its theoretical thickness (≈ 50 µm) for 10 mAh cm^−2^ lithium deposition, indicating a loose structure, whereas thickness of deposited Li with SIPIL10 is ≈ 70 µm (Figure 5j), indicating more compact structure. The digital photos of the surface of both deposited Li on copper and Li electrode also shows a remarkable difference in morphology. The deposited Li surface on copper appears less shiny (Figure S10a), and very dark Li surface morphology was detected on the Li counter electrode (Figure S10b) even after first plating that suggests lithium incompatibility, severe side reactions, and solvent degradation with the baseline electrolyte. Whereas Li metallic luster was clearly visible on both deposited Li surface on copper (Figure S10c) and Li counter electrode (Figure S10d), indicating SIPIL10 stabilizes the lithium metal interface and is compatible with it. The formation of high‐density compact nodule‐like lithium aggregates with larger particles and lower surface area successfully mitigates the electrode‐electrolyte interfacial reactions, resulting in high CE, stable cycle life, and enhanced safety of the lithium metal anode by SIPIL, everything required for the longevity of high performing LMBs.

### 2.4 Lithium Metal and Anodeless Cells with LFP Positive Electrodes and SIPIL Electrolyte

**FIGURE 6:**
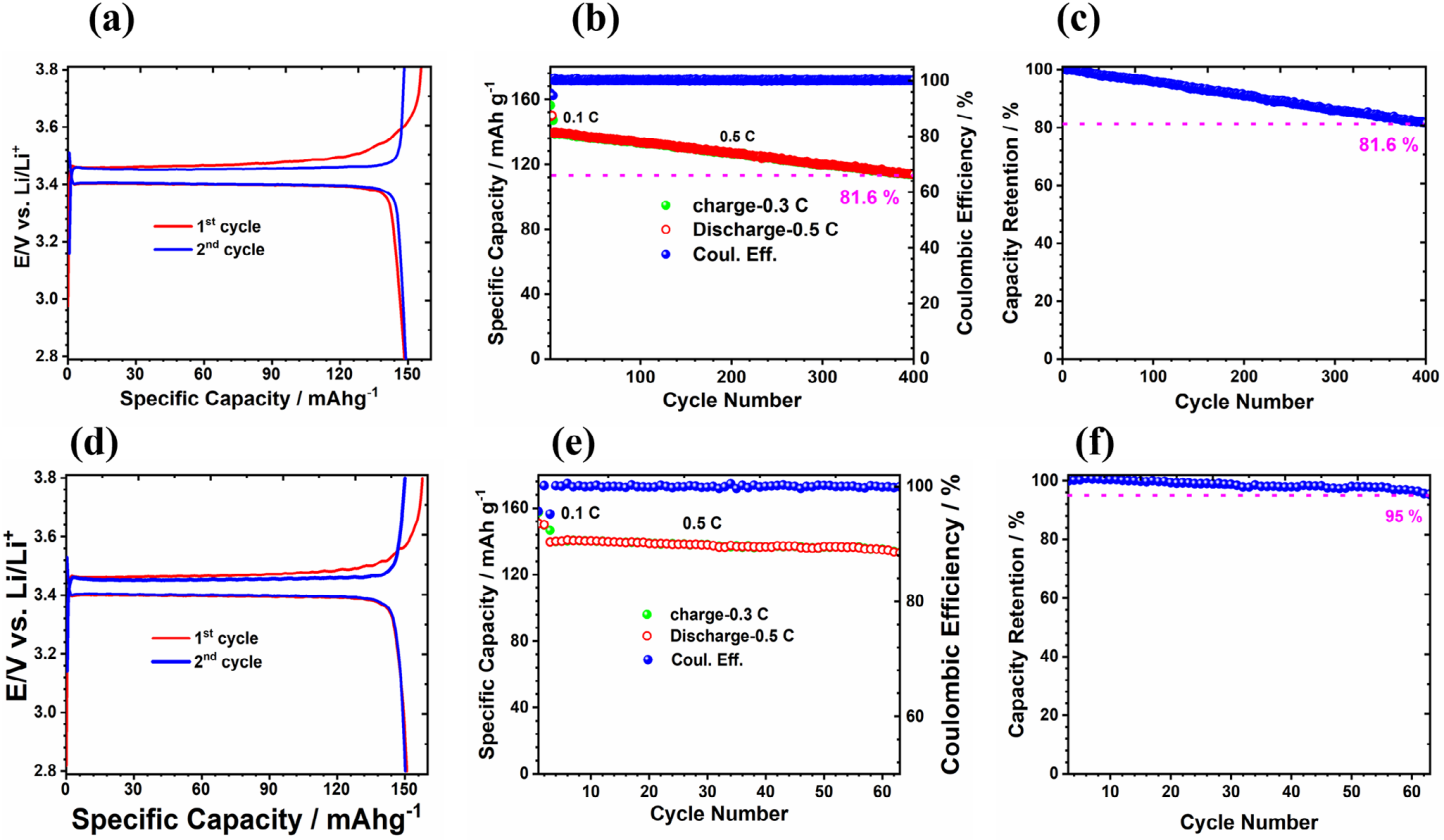
(a) First two charge–discharge voltage profiles of Li||LFP cell cycled at 0.1 C scan rate, (b) Specific (charge–discharge) capacities and coulombic efficiencies of Li|| LFP cell with SIPIL10 electrolyte as a function of cycle number from 2.8 – 3.8 V at 0.1 C for first three cycles, 0.3 C charge and 0.5 C discharge rate for the rest of the cycling period at RT, (c) Showing a CR of ≈ 81.6% for Li||LFP as a function of cycle number, with decay rate of 0.046% per cycle. (d) First two charge‐discharge voltage profiles of Cu||LFP cell cycled at 0.1 C scan rate, (e) Specific (charge–discharge) capacities and coulombic efficiencies of Cu|| LFP cell with SIPIL10 electrolyte as a function of cycle number from 2.8 – 3.8 V at 0.1 C for first three cycles, 0.3 C charge and 0.5 C discharge rate for the rest of the cycling period at RT, (f) Showing a CR of ≈ 95% for Cu||LFP as a function of cycle number, with decay rate of 0.07% per cycle.

Finally, the electrochemical performance of SIPIL10 was investigated in Li half‐cell configuration with Li as counter and reference electrode and LFP as working electrode (positive electrode) and Anodeless LMBs with copper as current collector and LFP as positive electrode. Both Li half and Anodeless LMB were cycled at 0.1 C (1 C = 170 mA g^−1^) for first three cycles and then charged at 0.3 C and discharged at 0.5 C for the following of the cycles. Figure 6a–c shows the charge discharge voltage profile, specific capacities and CE and capacity retention of Li||SIPIL10||LFP cell over 400 cycles. SIPIL10 exhibited first charge/discharge capacities of 156.3 / 149.28 mAh g^−1^ at a scan rate of 0.1 C, with CE of 95.5% and low overpotential of 52 mV (Figure 6a), indicating interfacial stability and compatibility with suppressed side reactions due to TFMSPyr induced robust SEI on Li electrode and fast lithium diffusion kinetics across the electrode‐electrolyte interface. After 400 cycles, SIPIL10 delivered a stable capacity of 113.8 mAh g^−1^ with capacity retention (CR) of 81.6%, corresponding to 0.046 decay rate per cycle (Figure 6b,c) with an average CE of ≈ 99.99%. It is worth noting that the CR was calculated compared with the first discharge capacity at 0.5 C (139.3 mAh g^−1^). When calculated using the first discharge capacity at a scan rate of 0.1 C (149.8 mAh g^−1^), CR is ≈76% after 400 cycles. Inspiring from the excellent electrochemical performance of SIPIL10 on both Li||LFP cell and Li||Cu cell, the electrochemical performance of SIPIL10 was investigated for Anodeless LMBs (Cu||LFP) using Cu as the current collector and LFP as the positive electrode. Figure 6d shows the charge discharge voltage profile for the first two cycles of the Cu||LFP cell. In Anodeless configuration, LFP with SIPIL10 electrolyte delivered first charge/discharge capacities of 157.6 / 150.8 mAh g^−1^ at a scan rate of 0.1 C, with CE of 95.7% and overpotential of 50 mV. The copper electrode (< 25 µm) was used as received without any treatment for removing oxide layer and surface smoothing. After 63 cycles, Cu||LFP cell delivered a stable capacity of 133.3 / 133.2 mAh g^−1^ with CR of 95% (calculated using first discharge capacity, 139.6 mAh g^−1^ at 0.5 C) corresponding to decay rate of 0.07 per cycle (Figure 6e,f), with an average CE of ≈ 99.2%. Such a high charge/discharge capacity with excellent lithium reversibility, CE, and CR indicate the suitability of SIPIL10 for Anodeless LMBs due to its capability to stabilize electrode‐electrolyte interface to form robust interphase. Feasibility of TFMSPyr as an electrolyte solvent for high‐voltage NMC811 cathode with cathode mass loading of ≈ 11 mg cm^−2^ was also investigated in Li||NMC811 half‐cell using 1.0 m LiTFSI salt. NMC811 cathode delivered first cycle discharge capacity of 199.5 mAh g^−1^ with CE ∼ 84% at 0.1 C (1 C = 200 mA g^−1^ is used for NMC811) and exhibits CR of ≈ 81% after 100 cycles (Figure S11a, b), whereas Li||NMC811 cells quickly failed with same concentration electrolyte in ethylene carbonate/ethyl methyl carbonate (3:7 by volume) because of aluminum current collector corrosion. These results indicate that TFMSPyr is stable, reduces current collector corrosion and promising electrolyte solvent for high‐voltage high‐capacity cathode with high active material loading for practical battery applications.

**FIGURE 7:**
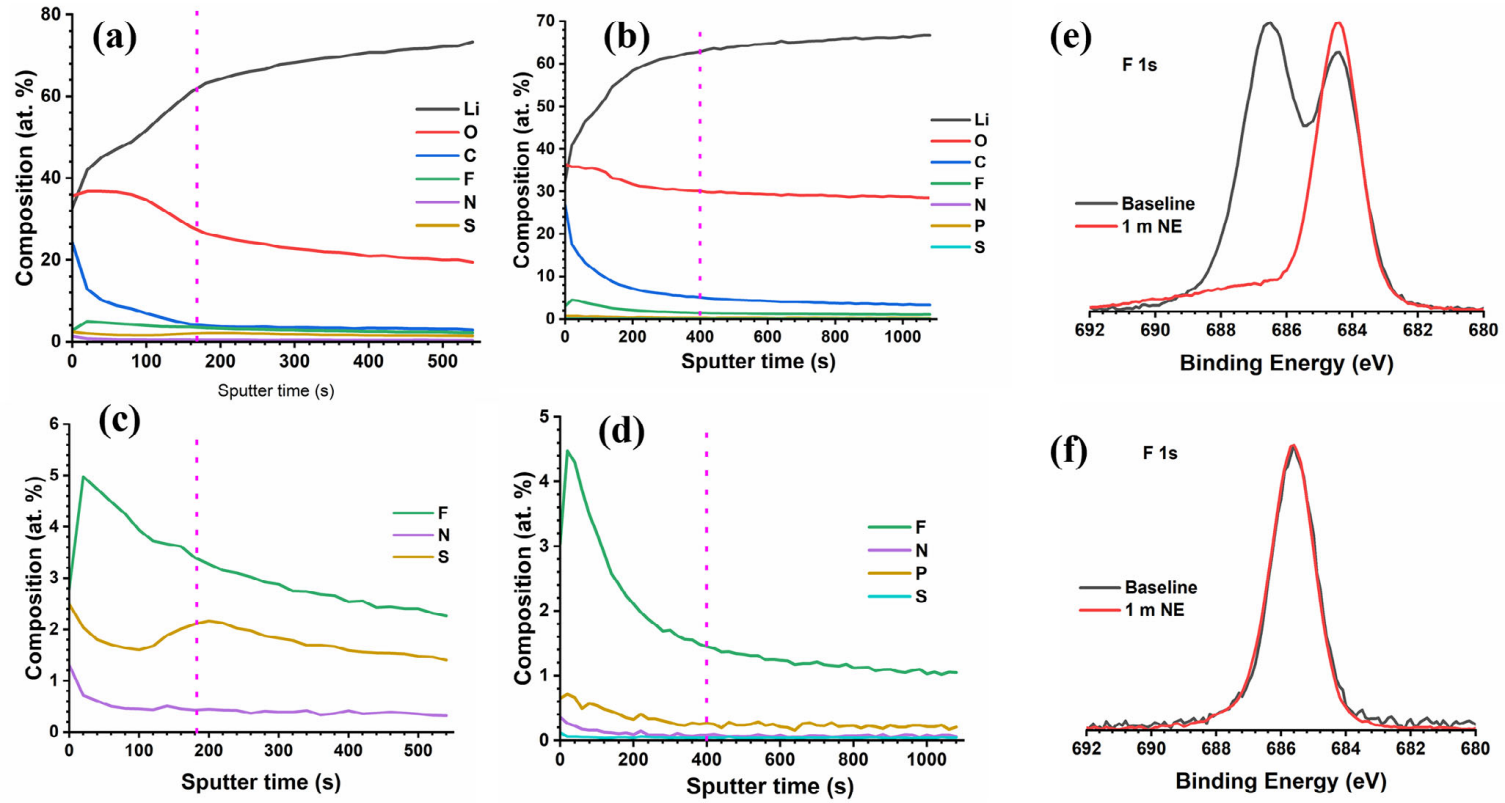
XPS depth profile measurements on Li electrode (a, c) for Li||SIPIL10||LFP and (b, d) for Li||Baseline||LFP after 3 cycles scan at 0.1 C rate from 2.8 – 3.8 V, showing CEI thickness as a function of sputtering time marked with dotted line. Core level XPS spectra of F 1s (e) before (f) after Ar^+^‐ion sputtering for depth profiling for baseline (black line) and SIPIL10 (red).

To elucidate the advantages of SIPIL10 over the baseline electrolyte in stabilizing the SEI on the negative electrode (Li), Li||LFP cell with both electrolytes were cycled at 0.1 C rate for first three cycles, during which SEI formation primarily occurs and subsequently stabilizes if no parasitic side reactions are present. The core level XPS spectrum of C 1s (Figure S12a) indicates that the baseline electrolyte produces a higher fraction of C‐F, C‐C and C/O (C = O & C‐O) species, along with more complex overlapping features, compared with SIPIL10. In contrast, O 1s and Li 1s spectra (Figure S12b, c) show no significant difference between the two electrolytes. The F 1s spectrum (Figure 7e) reveals a much higher amount of SEI component (C‐F, LiPO_x_F_y_) species (binding energy, B.E.≈688 eV) from the decomposition of LiPF_6_ and solvent for the baseline electrolyte, while SIPIL10 exhibits slightly higher inorganic‐F (Li‐F) species (B.E.≈685 eV) that was derived from the decomposition of anion from the anion dominated primary solvation shell. The SEI derived from preferential FSI anion decomposition was further validated by the DFT calculation in which S─F bond of FSI spontaneously breaks whereas no bond breakage was observed in TFMSPyr, suggesting TFMSPyr is more stable compared to FSI. Exact quantification of the transition state pathways requires searching through many different combinations of bond‐breakage which is beyond the scope of the current study (see supporting information and Figure S13 for more information).

After Ar‐etching (Figure 7f), both electrolytes show comparable amounts of inorganic‐F (Li‐F) species, suggesting that most SEI components were removed during the depth profiling leaving only LiF signal formed on the Li surface. This observation indicates that the higher content of the carbon species in the baseline electrolyte originates from carbonate decomposition, which is less stable than TFMSPyr under the highly reductive environment at Li metal.

The SEI thickness was evaluated by XPS depth profiling through Ar^+^‐ion‐ion etching of Li electrodes. The evolution of SEI was tracked based on the progressive increase in Li signal intensity and the corresponding decrease in F‐containing species as a function of sputtering time. For SIPIL10, the concentration of Li and f species reached a steady state after approximately 200 s of sputtering time (Figure 7a,c), whereas the baseline electrolyte required about 400 s to reach a comparable profile (Figure 7b,d). This result indicates that the SEI formed in the SIPIL10 electrolyte is approximately half as thick as that produced with the baseline electrolyte.

The thinner SEI in the SIPIL10 system demonstrates its superior compatibility with Li and ability to regulate interphase growth by suppressing excessive electrolyte decomposition and promoting the formation of a compact, inorganic‐rich passivation layer. Such controlled SEI formation minimizes interfacial impedance and enhances li‐ion transport kinetics, ultimately contributing to improved cycling stability and coulombic efficiency. It should be noted, however, that the precise quantification of SEI thickness from XPS depth profiling remains approximate due to potential sputtering‐induced artifacts and compositional heterogeneity within the interphase.

**FIGURE 8:**
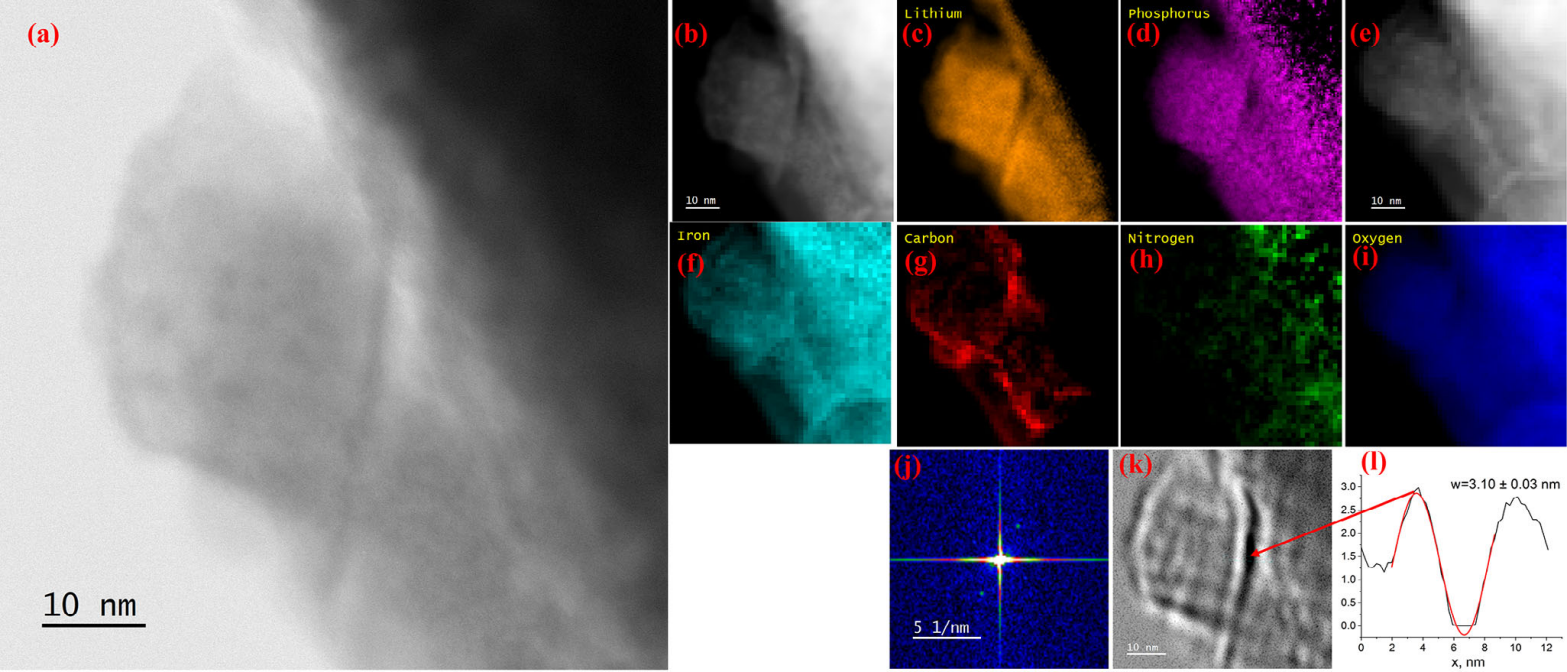
HR‐STEM images of cycled LFP electrode after 3 cycles in SIPIL10. (a) ABF image, (b) ADF image acquired simultaneously with EELs data for the lower energy range with (c) Li K and (d) P L edges, (e) ADF image acquired simultaneously with EELs data for the higher energy range with (f) Fe L, (g) C K, (h) N K, and(i) O K edges. CEI thickness measurement from NMF decomposition of HAADF images. (j) ABF image of cycled LFP particles, (j) Component 2 of a 4‐component NMF decomposition of the ABF image, (k) mixing coefficient map corresponding to component 2 with a profile region indicated by a blue rectangle, (l) profile with the corresponding sine fit and width measurement showing the approximate thickness of CEI.

To investigate the growth of cathode electrolyte interphase (CEI) of the cycled LFP cathodes in baseline and SIPIL10 electrolyte, the surface of the cycled electrode (after 3 charge‐discharge cycles) was probed by STEM‐EELS. For cycled LFP with SIPIL 10 electrolyte, all elements (Fe, Li, P, C, N, O) are distributed homogeneously within the LFP particles (Figure 8b‐i; Figure S14 and Table S5) with slightly lower carbon content compared with LFP cycled with baseline electrolyte (Figures S15b‐i; S16, and Table S6), indicating greater stability of TFMSPyr solvent. To visualize the CEI thickness differences in the images, we used diffractograms of Annular Bright Field (ABF) image patches to identify structurally distinct regions at the particle edges. A sliding window of 256×256 pixels was used to generate a 2D array of fast Fourier transforms. Non‐negative matrix factorization (NMF) approach was then used to analyze the 4D dataset. This analysis helps identify and visualize amorphous and crystalline regions with distinct structural signatures. Across this system, the crystalline regions appear to have consistent lattice parameters, with no localized regions of altered crystal structure being identified; however, the edge sharpness of these regions varies, which we attribute to the CEI. Figure 8j–l shows the ABF STEM image of cycled LFP in SIPIL10 electrolyte, followed by component 2 and the corresponding coefficient map of 4‐component NMF decomposition of HAADF image. The profiles across the particle edge, or CEI, (denoted by blue rectangular box on the coefficient map) show a thickness of ≈ 3.1 nm, which is lower than the CEI thickness on cycled LFP with baseline electrolyte (CEI thickness ≈ 4.54 nm, Figure S15j–l), indicating the lesser parasitic side reaction at cathode‐electrolyte interface with SIPIL10 as a result of electrochemically stable TFMSPyr (See Figures S17 and S18 for the full NMF decomposition for cycled LFP cathodes with SIPIL10 and baseline electrolytes, respectively).

SIPIL10 exhibited significantly improved electrochemical performance compared to baseline electrolytes across various cell configurations. In both Li||Li and Li||Cu cells, SIPIL10 enabled highly stable cycling with prolonged lifespan and elevated plating/stripping CE, indicative of uniform Li nucleation and suppressed dendritic growth. SIPIL10 demonstrated exceptional electrochemical performance compared with the state‐of‐art WSE reported in the literature (see supporting information Table S7). These improvements can be attributed to the formation of compact, inorganic‐rich SEI and CEI facilitated by the tailored solvation structure of TFMSPyr and LiFSI with weak Li^+^‐TFMSPyr and AGGs formation in SIPIL10, which effectively mitigates parasitic reactions and uncontrolled interphase growth. Wider electrochemical stability window and a higher Li^+^ transference number of SIPIL10 enable more efficient charge transport and improved interfacial kinetics. Consequently, SIPIL10 delivered superior electrochemical performance in both Li||LFP and Anodeless Cu||LFP LMBs, where the controlled interfacial chemistry stabilized the electrode/electrolyte interface and minimized electrolyte depletion, leading to enhanced CE, cycle life, and overall cell reversibility. This encouraging electrochemical performance supports the future large‐scale production of TFMSPyr, which reduces the cost (the current raw material costs are listed in Table S8) of TFMSPyr electrolyte for battery applications.

## 3 Conclusions

In summary, we introduced a rational molecular engineering approach to design a weakly‐solvating TFMSPyr molecule, a PIL that represents a new design solvent design paradigm, reproducing ionic liquid like electrostatic solvation through intramolecular charge separation. This strategy enables weakly solvation environment and improved ion transport while overcoming the viscosity limitations typically associated with ionic liquids. Thus, formulated SIPIL effectively stabilizes the Li‐electrolyte interface through the formation of a thin, inorganic‐rich SEI with controlled composition and morphology. Anion dominated solvation structure and weak interactions of TFMSPyr with Li‐ion resulted in CIP/AGGs formations suppressing parasitic reactions, leading to high CE, wide electrochemical stability, and improved Li‐ion transport. Overall, the LiFSI–TFMSPyr system exemplifies a rationally designed electrolyte in which molecular architecture, solvation topology, and transport dynamics are intrinsically coupled. These interfacial advantages translate into exceptional electrochemical performance across multiple cell configurations, including Li||Li, Li||Cu, Li||LFP, and Anodeless Cu||LFP cells, demonstrating prolonged cycling stability and superior reversibility. For example, Li||Cu cells deliver a first cycle Coulombic efficiency (FCCE) of ≈ 99% and maintain ≈ 99.2% coulombic efficiency on average, with 82% capacity retention after 400 cycles in Li||LFP half‐cell test. In Anodeless full cells, 95% of initial capacity is retained after 63 cycles with an average CE of 99.5%. This work bridges the gap between localized high‐concentration and ionic‐liquid electrolyte chemistries and highlights the critical role of molecular‐level solvent engineering, offering a blueprint for developing advanced energy materials that unite high stability, safety, and cation‐selective transport for achieving durable interfacial chemistry for future Anodeless/ LMBs and high‐voltage batteries.

## 4 Experimental Section

### 4.1 Materials and Methods

Trifluoromethanesulfonyl chloride (Sigma Aldrich), triethylamine (Sigma Aldrich), Pyrrolidine (Fischer), dichloromethane (Sigma Aldrich), hydrochloric acid (Sigma Aldrich), anhydrous magnesium sulphate (Fischer), and battery grade lithium bis(fluoromethanesulfonyl)imide (Sigma Aldrich) were used as received.

### 4.2 Preparation of 1‐trifluoromethanesulfonyl pyrrolidine (TFMSPyr)

13.62 g of pyrrolidine (0.1765 mol) and 17.865 g of triethyl amine (0.1765 mol) were added to the 500 mL two neck round bottom flask. 100 mL anhydrous dichloromethane was added to the flask and cooled at −6°C in an ice acetone bath. Then, 29.74 g of trifluoromethanesulfonyl chloride (0.1765 mol) was added dropwise via a dropping funnel with vigorous stirring. Followed by addition of trifluoromethanesulfonyl chloride, the reaction mixture was allowed to reach the room temperature and stirred at this temperature overnight. After the completion of the reaction, 100 mL 1 M HCl was added to the reaction mixture and organic phase was separated using separating funnel and washed with brine solution twice (2 × 50 mL). The washed organic phase was dried using anhydrous magnesium sulfate and filtered followed by rotary evaporation to remove residual solvent. The resulting product was named as 1‐trifluoromethanesulfonyl pyrrolidine (TFMSPyr), hereafter, referred as TFMSPyr. Finally, TFMSPyr was purified via distillation to get colorless liquid and stored in Ar filled glove box (oxygen and moisture less than 0.5 ppm). The purity of the TFMSPyr was ≈ 99. 9% after distillation (determined by proton NMR spectroscopy), with final recovered yield of the anhydrous TFMSPyr was ≈ 85% with reproducibility verified by multiple batch synthesis (5 different batches). The structure of the TFMSPyr was confirmed by ^1^HNMR, ^13^CNMR, and ^19^FNMR. Battery grade lithium bis(fluoromethanesulfonyl)imide was dissolved in TFMSPyr to make 0.5 m and 1.0 m solution, hereafter referred as “SIPIL5” and “SIPIL10” respectively, for further characterization. 1.2 M LiPF_6_ in ethylene carbonate/ethylmethyl carbonate (EC/EMC) (3:7 volume ratio) was used as baseline electrolyte and hereafter referred as “Baseline electrolyte”.

### 4.3 Material Characterization

^1^H, ^13^C, and ^19^F NMR of the synthesized TFMSPyr were recorded at room temperature using a JEOL 400 YH spectrometer (400 MHz) with DMSO‐*d_6_* as a solvent (spectra shown in supporting information). The reported chemical shifts were referenced to the residual solvent peak. A Bruker 400 MHz instrument was used to perform Pulse‐field gradient NMR (PFG‐NMR) experiments to track the diffusion of Li^+^ and FSI^−^ ions, and TFMSPyr solvents by monitoring Li and F atom [37]. The gradient range was chosen to observe significant decay in intensity. TopSpin 4.4.0 software (Bruker) was used to process the data, and the relative intensity was fit to a nano‐disperse exponential decay [53].

(1) $$\frac{I}{I_{0}}=\exp\left[-D{\left(\gamma G\delta \right)}^{2}\left(\Delta -\frac{\delta }{3}\right)\right]$$

where *I/I_0_* corresponds was the relative intensity, *G* was the gradient applied, *∆* was the diffusion time, δ was the duration of gradient pulse, γ was the gyromagnetic ratio and *D* was the diffusivity of atom being tracked. These self‐diffusivity data were used to calculate the lithium‐ion transport number. Raman spectra were measured using Raman spectroscopy (with a 532 nm laser wavelength) by a Renishaw inVia confocal Raman microscope. The cycled lithium electrodes were loaded into the XPS vacuum transfer module from Ar‐filled glove box, transfer to XPS load‐lock, and then moved into the XPS analysis chamber without air exposure. A wide energy range survey spectrum was acquired for each electrode sample to identify all the solid electrolyte interphase (SEI) components present, followed by a collection of narrow energy range core level spectra for each identified species. Surface morphology and cross‐sectional analysis of the deposited lithium on copper was performed on a ThermoFisher Helios 5 Hydra CX plasma focused ion beam instrument (P)FIB fitted with a Quorum PP3010 cryogenic system. The lithium deposited copper electrode was hermetically sealed in a plastic tube in a glove box and transported to the (P)FIB instrument. The plastic tube was cooled and subsequently opened under liquid nitrogen and then transferred into the (P)FIB under a low vacuum while maintaining cryogenic conditions. The sample was held at − 192°C during imaging and PFIB work. Top‐down backscattered electron (BSE) surface images were acquired at 2 kV from both baseline and 1 m LiFSI‐TFMSPyr samples. Trenches were cut into the samples to image the cross‐section and calculate the thickness of the deposited lithium. The surface compositions on the cycled LFP cathodes were studied using high resolution scanning transmission electron microscopy (HR‐STEM). Elemental distribution was characterized by the Nion ULTRASTEM 100 microscope operated at 100 kV and equipped with a Dectris ELA EEL spectrometer. Spectra were collected in two energy ranges for each area to allow for sufficient resolution to cover all elements of interest; edges appearing in both ranges were used to check the consistency of elemental quantification. EEL spectrum images were analyzed using the Gatan software suite with corrections for peak overlap and multiple scattering.

### 4.4 Quasielastic Neutron Scattering (QENS)

Molecular‐level dynamics of cyclic sulfonamide and its 1 m solution with LiFSI were explored using the backscattering silicon spectrometer (BASIS) [54] at the Spallation Neutron Source of Oak Ridge National Laboratory. QENS spectra were collected using incident neutrons with a wavelength band centered at 6. 4 Å, probing the energy transfer (E) range of ± 100 µeV and the momentum transfer (Q) range of 0.2 –2.0 Å^−1^ by making use of Si 111 analyzer panels that backscatter the neutrons scattered from the samples to the detectors. Annular aluminum sample cans with inserts providing a 0.1 mm gap for the samples were used. An indium wire was used to seal the sample cans. The samples were prepared and loaded inside a glovebox to avoid contamination from the atmospheric moisture. QENS spectra at 300 K and the sample‐specific resolution function at 30 K were measured from each sample by controlling the temperature using a closed cycle refrigerator (CCR). The data reduction was done using Mantid [55], and the analysis was performed using DAVE [56] software. The total measured QENS signal, *I(Q, E)*, which was the sum of the contributions from the elastic, quasielastic, and inelastic neutron scattering events, was given by: [40, 57]

(2) $$\begin{array}{ccc} I\left(Q,E\right) & = & \left[X\left(Q\right)\delta \left(E\right)+\left(1-X\left(Q\right)\right)S\left(Q,E\right)\right]⊗R\left(Q,E\right)\\ & & +\;B\left(Q,E\right) \end{array}$$

The fraction of elastic contribution, *X*(*Q*), was described by a delta function, δ(E). Since the motion faster than the sensitivity limit of the instrument was a part of a linear background term, *B*(*Q*, *E*), the measurable quasielastic contribution from the mobile species in the samples was described by the dynamic structure factor, *S*(*Q*, *E*). The *S*(*Q*, *E*) was modeled on a sum of two Lorentz functions. After numerical convolution (⊗) with the instrument resolution function, *R*(*Q*, *E*), the half widths at half maximum (HWHMs) of those Lorentzians were extracted from the model scattering function:

(3) $$S\;\left(Q,E\right)=p\left(Q\right)\frac{1}{\pi }\frac{\Gamma_{1}\left(Q\right)}{\Gamma_{1}^{2}\left(Q\right)+E^{2}}+\left(1-p\left(Q\right)\right)\frac{1}{\pi }\frac{\Gamma_{2}\left(Q\right)}{\Gamma_{2}^{2}\left(Q\right)+E^{2}}$$

In Equation (3), Γ_1_(*Q*) and Γ_2_(*Q*) were the HWHMs of the first and second Lorentzian functions representing the slow and fast dynamics processes measurable in the samples. The terms *p*(*Q*) and 1‐ *p*(*Q*) denote the relative spectral weights of first and second Lorentzians. Q‐dependence of the slow component, representing long‐range translational diffusion, was fitted using Fickian diffusion model as *HWHM*(*Q*)  =  ℏ*DQ* ^2^, where D was the self‐diffusion coefficient and ℏ was the reduced Planck's constant. QENS signals were predominantly sensitive to hydrogen‐bearing species, such as solvent molecules in neat solvents or salt solutions.

### 4.5 Simulation Methodology

*Quantum Mechanical Simulations*: Quantum mechanical simulations were carried out in Gaussian‐16. A density functional theory calculation using B3LYP functional and 6–31+G(d, p) basis sets were used to optimize the structure of TFMSPyr, EC and EMC molecules in vacuum. The initial structures and the naming conventions of these molecules were given in Figure S19.

*Molecular Dynamics Simulations*: We used all‐atom molecular dynamics simulations to understand the solvation structure of LiFSI in TFMSPyr and EC:EMC (3:7 by weight) solutions. Simulations were performed using GROMACS‐v2024.5 software [58].

The Lennard‐Jones and Coulombic interaction parameters for lithium cations and FSI anions were modeled using CL&P force fields [59, 60]. The Lennard‐Jones parameters for these TFMSPyr, EC and EMC were obtained from LigParGen software. To compute the charges on these molecules, we employed the RESP method with Multiwfn software [61] using the optimized structures obtained from density functional theory calculations. All the charges in the system were scaled by a factor of 0.8 to account for polarizability [62]. Van der Waal's interactions between different atom types were modeled using Lorentz‐Berthelot mixing rules. In addition, the 1–4 intramolecular Lennard‐Jones and electrostatic interactions were scaled by a factor of 0.5. The Lennard‐Jones and charge parameters were provided in Table S9.

The initial configurations of TFMSPyr, EC and EMC were generated using Avogadro software [63] the those for FSI molecules were generated using Avogadro and fftool [64] software. These molecules were subsequently packed in a cubic box with equivalent lithium cations at a density of approximately 1.0 g/cc using PACKMOL software [65]. The initial box dimensions and the number of chains of each type were given in Table S10.

An energy minimization run with steepest descent was initially conducted. Subsequently, an NVT run using modified Berendsen thermostat and a time constant of 0.1 ps was used for equilibration at 300 K for 10 ns. Each of the molecule types (Li, FSI and TFMSPyr) were coupled to separate temperature‐coupling baths. Following the NVT equilibration protocol, an NpT protocol with C‐rescale barostat [66] and a modified Berendsen thermostat was used to run the system at 1 bar pressure and a temperature of 300 K for 10 ns. The thermostat and barostat coupling constants were set at 0.1 ps and 4 ps, respectively. The production cycle comprised of another 100 ns in the NpT ensemble with a Parinello‐Rahman barostat [67] at 1 bar pressure and a modified Berendsen thermostat at 300 K. The thermostat and barostat coupling constants were 0.2 ps and 8 ps, respectively. Periodic boundary conditions were applied to all three dimensions. Hydrogen atoms were constrained using the LINCS algorithm [68]. A leap‐frog integrator with a timestep varying between 0.5 and 2 fs were used to integrate the equations of motions. The cut‐off distances for both the electrostatics and van der Waal's interactions were set at 1.0 nm. Particle‐Mesh‐Ewald (PME) [69] was used to compute the long‐ranged electrostatics, and the error tolerance was set at 1 × 10^−5^.

The forcefields were validated by comparing the density for 1.0 m LiFSI in TFMSPyr solvents. Simulations yielded a density of 1.49 g/cc and were consistent with the experimental observations (≈1.4 g/cc). All the results shown were averaged over the last 18 ns of two independent initial simulations.

The GROMACS simulation files, PACKMOL scripts, topology files and interaction potential files were available at https://github.com/vaidyanathanms/MD_LiSalts.git.

### 4.6 Electrochemical Characterization

The ionic conductivity of SIPIL10 (1 m LiFSI in TFMSPyr) was measured by electrochemical impedance spectroscopy (EIS) on Bio‐logic VSP over frequency ranging from 200 KHz to 100 MHz at room temperature. The custom‐made conductivity cell with two parallel electrodes made of platinum discs was used for the conductivity measurement. Standard 0.1 M KCl solution was used to determine the cell constant of the conductivity cell used in this work. The electrochemical potential stability window was measured via cyclic voltammetry (CV) scanning from −0.25 V– 3.00 V versus Li/Li^+^ at a scan rate of 1 mV s^−1^ and linear sweep voltammetry (LSV) scanning from 2.5 V to 6.0 V versus Li/Li^+^ at a scan rate of 10 mV s^−1^ in a coin cell with Li|| SIPIL10||Stainless steel (SS) two electrode setup. The lithium transference number (*t*_Li+_) was calculated using the following equation, known as Bruce‐Vincent method [70, 71], involving DC polarization and AC impedance measurements of symmetric Li||SIPIL10||Li cells and compared with the value obtained from PFG‐NMR method.

(4) $$t_{Li^{+}}=\frac{\left[I_{s}\left(\Delta V-I_{i}R_{i}\right)\right]}{\left[I_{i}\left(\Delta V-I_{s}R_{s}\right)\right]}\;$$

where Δ*V* was the polarization bias voltage, and *I*_i,_ *R*_i_, *I*_s_, and *R*_s_ were initial and steady‐state currents and resistances, respectively.

The interfacial stability of this new electrolyte was investigated by lithium plating‐stripping cycles on symmetric Li||SIPIL10||Li cell in coin cell configuration, operating periodically plating 1 mAh cm^−2^ Li at a rate of 0.5 mA cm^−2^ current density for 2 h followed by 10 min rest and stripping the plated lithium at the same current density for 2 h. Different current density scans from 0.5, 1.0, and 2 mA cm^−2^ were conducted to determine the limiting current density. The cycling stability and lithium plating‐stripping coulombic efficiency was measured in Li||Cu half‐cell using SIPIL10 electrolyte. All the coin cells were assembled in the glove box with Li disc as counter and reference electrode and Cu as a working electrode for lithium plating substrate. It was worth to note that the Cu foil (≈ 20 µm thickness) used in this work was used as received without further treatment. The current density for the lithium metal plating/stripping was set to 0.5 mA cm^−2^ using Land battery testing at room temperature. The effective area of Cu foil for Li deposition was 1.27 cm^−2^. During each cycle, 1, or 2.5 mAh cm^−2^ of Li metal was deposited on the Cu foil either for 2 or 5 hrs plating time followed by stripping Li metal until 1.0 V potential versus Li/Li^+^. The Li plating/stripping efficiency was also calculated by the Aurbach method to eliminate the uncertainty related to the substrate surface. In this method, Li was plated on Cu foil depositing 5 mAh cm^−2^ of Li for 10 hrs at a rate of 0.5 mA cm^−2^ followed by Li striping until the potential reached to 1.0 V. In the subsequent cycle, 5 mAh cm^−2^ of Li was plated for 10 hrs at the same rate followed by stripping of 0.5 mAh cm^−2^ of Li for 1 hr. For the next 20 cycles, the plating/stripping was conducted at 0.5 mA cm^−2^ rate for 1 h to plate and strip 0.5 mAh cm^−2^ of Li followed by stripping of Li until potential reached to 1.0 V at the last stripping cycle.

Lithium iron phosphate (LiFePO_4_, LFP) cathodes were prepared by casting a homogeneous slurry of LFP, carbon black (CB 45), and PVDF in N‐methyl‐pyrrolidone (NMP) onto aluminum foil, followed by drying under vacuum at 110°C. The weight ratio of LFP, CB45, PVDF was 80:10:10. Circular discs with electrode surface areas of 1.27 cm^2^ and active mass loadings of ≈1.0‐1.3 mg cm^−2^ for LFP were punched out. All electrochemical tests were conducted in 2032‐type coin cells assembled with lithium anodes, LFP cathodes, and SIPIL10 as electrolytes and separators in an argon‐filled glove box (oxygen and moisture < 0.5 ppm). The electrochemical performance of Cu||SIPIL10||LFP cells were used to analyze the suitability of SIPIL10 for the Anodeless LMBs. Battery performance testing was carried out using an Arbin Battery testing instrument, with cyclic voltammetry (CV) performed on a Bio‐logic VSP instrument. Galvanostatic charge–discharge cycles for LFP were executed over a voltage range of 2.8 –3.8 V at a scan rate of 0.1 C for the initial three cycles and charge/discharge at a scan rate of 0.3 / 0.5 C at RT for both Li||LFP half cells and Cu||LFP Anodeless LMBs for the remainder of the cycles.

## Conflicts of Interest

The authors declare no conflict of interest.

## Supporting information




**Supporting file**: advs75550‐sup‐0001‐SuppMat.docx

## Data Availability

The data that supports the findings of this study are available in the supplementary material of this article.
