# Supplementary material for: Rational Design of Weakly‐Solvating Molecules for Salt‐In‐Pre‐Ionic‐Liquid Electrolytes for Li Metal Batteries
Source: Adv Sci (Weinh). 2026 May 7;13(42):e75550. doi: 10.1002/advs.75550 (PMC13335877; doi:10.1002/advs.75550)
Supplement: Supplementary file 1 — Supporting File: advs75550‐sup‐0001‐SuppMat.docx. [file ADVS-13-e75550-s001.docx]

Supporting Information

**Rational Design of Weakly-Solvating Molecules for Salt-In-Pre-Ionic-Liquid Electrolytes for Li Metal Batteries**

*Bishnu P. Thapaliya^*^, Vaidyanathan Sethuraman, Naresh C. Osti, Arvind Ganesan, K Shawn Reeves, Michael J Zachman, Albina Y Borisevich, Harry M Meyer III, Xiao-Guang Sun, Eugene Mamontov, Lei Cheng, Sheng Dai^*^*

Dr. Bishnu P. Thapaliya, Dr. Vaidyanathan Sethuraman, Dr. Arvind Ganesan, Dr. Harry M Meyer III, Dr. Xiao-Guang Sun, Dr. Lei Cheng, Dr. Sheng Dai

Chemical Sciences Division, Oak Ridge National Laboratory, Oak Ridge, TN 37831, United States

*^*^E-mail:* [*prasadthapab@ornl.gov*](mailto:prasadthapab@ornl.gov)*,* [*dais@ornl.gov*](mailto:dais@ornl.gov)

Dr. Naresh C Osti, Dr. Eugene Mamontov

Neutron Scattering Division, Oak Ridge National Laboratory, Oak Ridge, Tennessee 37831, United States

K Shawn Reeves, Dr. Michael J Zachman, Dr. Albina Y Borisevich

Center for Nanophase Materials Sciences, Oak Ridge National laboratory, Oak Ridge, TN 37831, USA

| 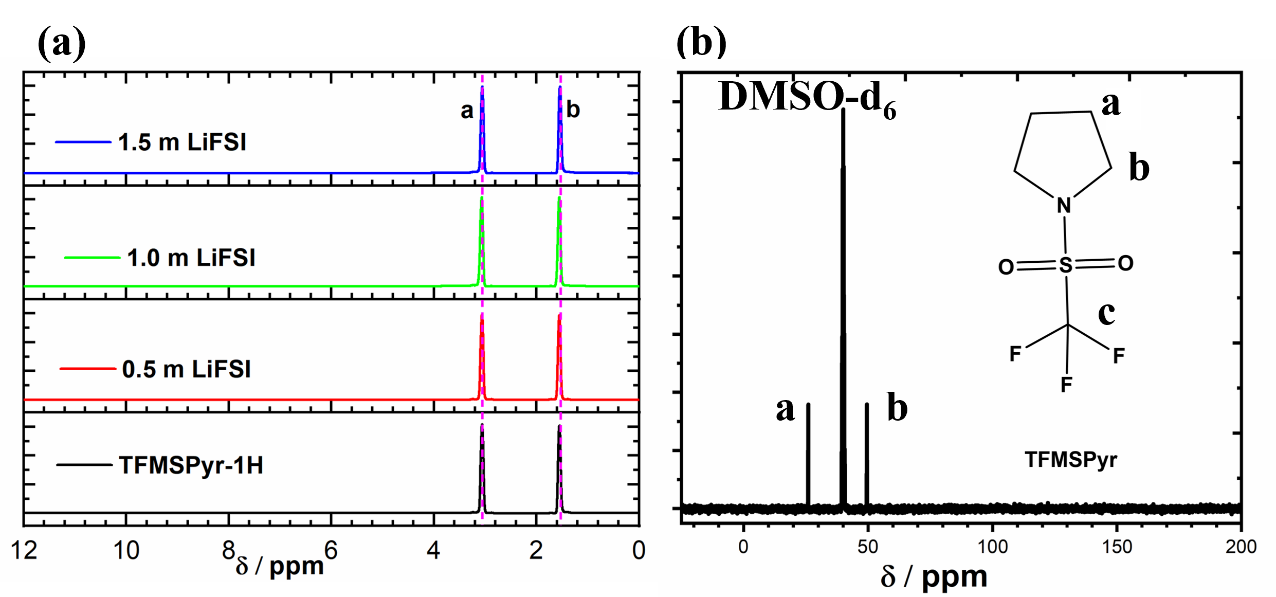 |
| --- |

Figure S1. (a) ^1^H NMR spectra of TFMSPyr and different concentrations of LiFSI in TFMSPyr in CDCl_3_, (b) ^13^C NMR spectrum of TFMSPyr DMSO-d_6_. Inset figure in (b) shows the molecular structure of TFMSPyr with a, b, and c representing types of hydrogen and carbon. TFMSPyr has two types of hydrogen and 3 types of carbon. CF_3_ carbon is not seen in the spectrum in b because of the lower concentration and the shorter scan rate.

Table S1. The viscosity of TFMSPyr, SIPIL10, methylpropylpyrrolidinum-bis(Trifluoromethanesulfonyl)imide (MPPyr-TFSI) and 1.0 m LiFSI in MPPyr-TFSI

| Sample Name | Viscosity (cP) |
| --- | --- |
| TFMSPyr | 3.85 |
| SIPIL10 | 16.2 |
| MPPyr-TFSI | 67.1 |
| 1.0 m LiFSI in MPPyr-TFSI | 307.1 |

|  |
| --- |


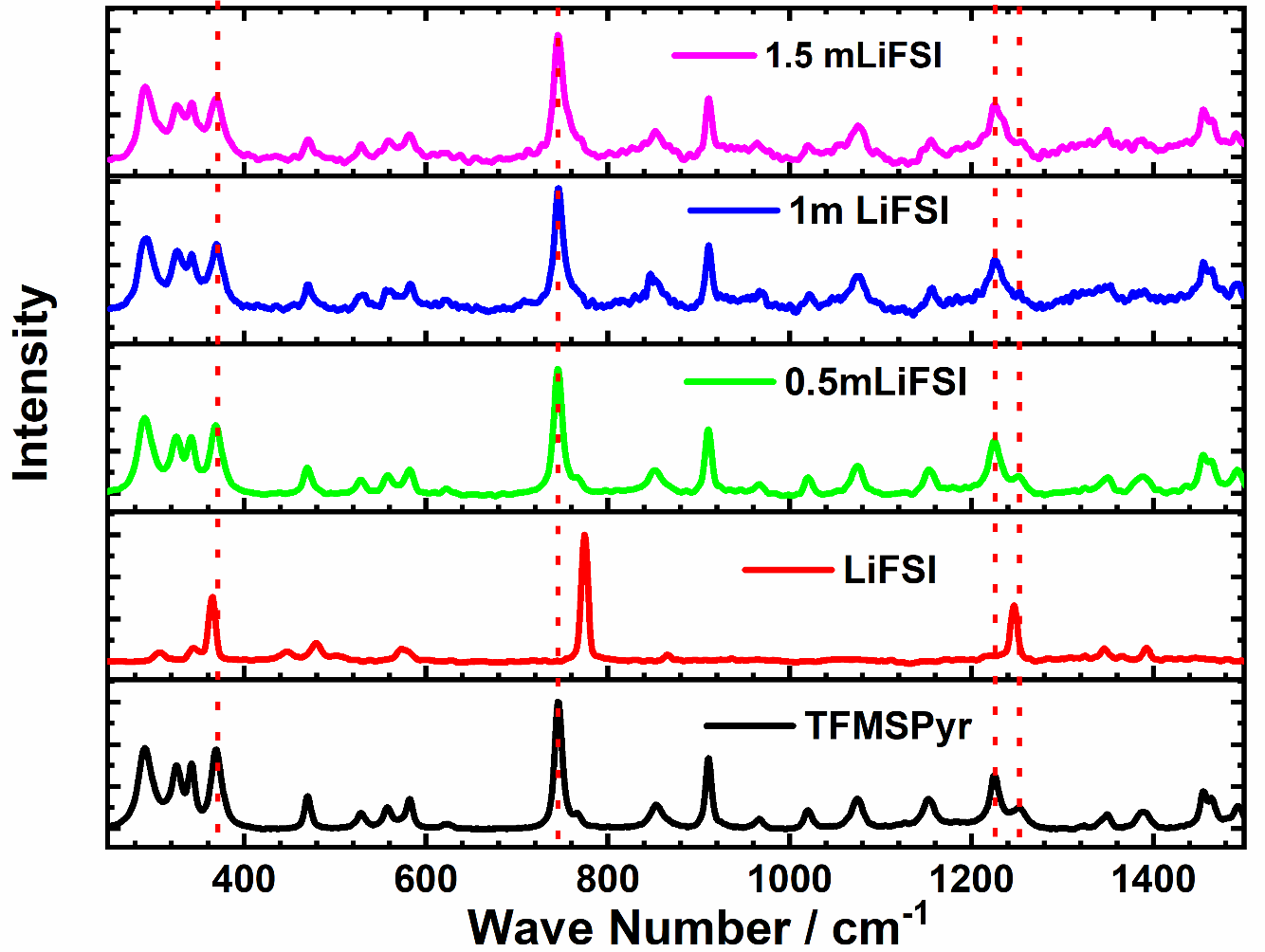


Figure S2. Raman spectra of TFMSPyr and different concentrations LiFSI in TFMSPyr.


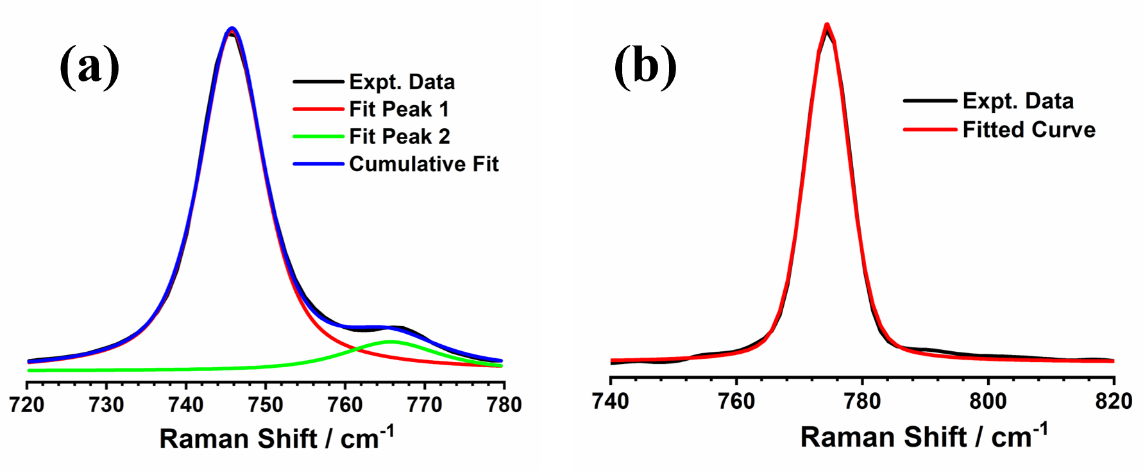


Figure S3. Raman spectra of (a) TFMSPyr and (b) LiFSI from 720 to 780 cm^-1^

**Table S2**: Fitted Raman spectrum of different concentration of LiFSI in TFMSPyr from 720 – 780 cm^-1^ range showing the peak positions of CIPs, AGGs and Solvents

| Conc. | CIPs/ cm^-1^ | S-N-S peak of TFMSPyr/ cm^-1^ | AGGs / cm^-1^ |
| --- | --- | --- | --- |
| 0.5 m | 742.2 | 745.4 | 747.8 |
| 1.0 m | 743.0 | 745.8 | 752.8 |
| 1.5 m | 743.4 | 746.6 | 757.6 |

**Table S3**: Lithium-transference number (*t*_Li_^+^) measured by NMR and EIS

| Conc. of LiFSI (m) | δ_solvent_ / m^2^ s^-1^ | δ_FSI_^-^ / m^2^ s^-1^ | δ_Li_^+^ / m^2^ s^-1^ | *t*_Li_^+^ (NMR) | *t*_Li_^+^ (EIS) |
| --- | --- | --- | --- | --- | --- |
| 0.0 m LiFSI | 3.01E-10 | - | - | - | - |
| 0.5 m LiFSI | 1.63E-10 | 0.81E-10 | 0.85E-10 | 0.51 | - |
| 1.0 m LiFSI | 0.88E-10 | 0.42E-10 | 0.44E-10 | 0.51 | 0.61 |
| 1.5 m LiFSI | 0.45E-10 | 0.21E-10 | 0.23E-10 | 0.52 | - |

| 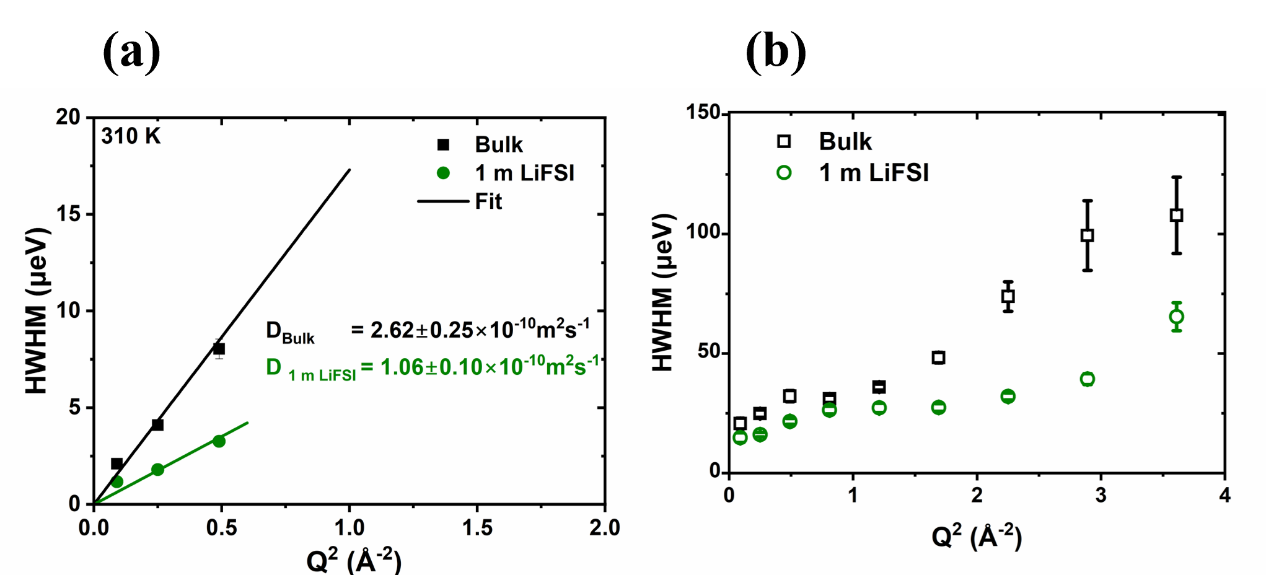 |
| --- |

Figure S4. (a) A comparison of the Q- dependence of the half width at total maximum (HWHM) was obtained from the model fit as a function of the square of the momentum transfer vector of the bulk and the 1 m LiFSI salt solution in cyclic sulfonamide. HWHM of the narrow component fitted with $HWHM=\hbar Dq^{2}$ law. Diffusion coefficients obtained are also shown, (b) Dependence of the HWHM of the broad component with square of Q.

| 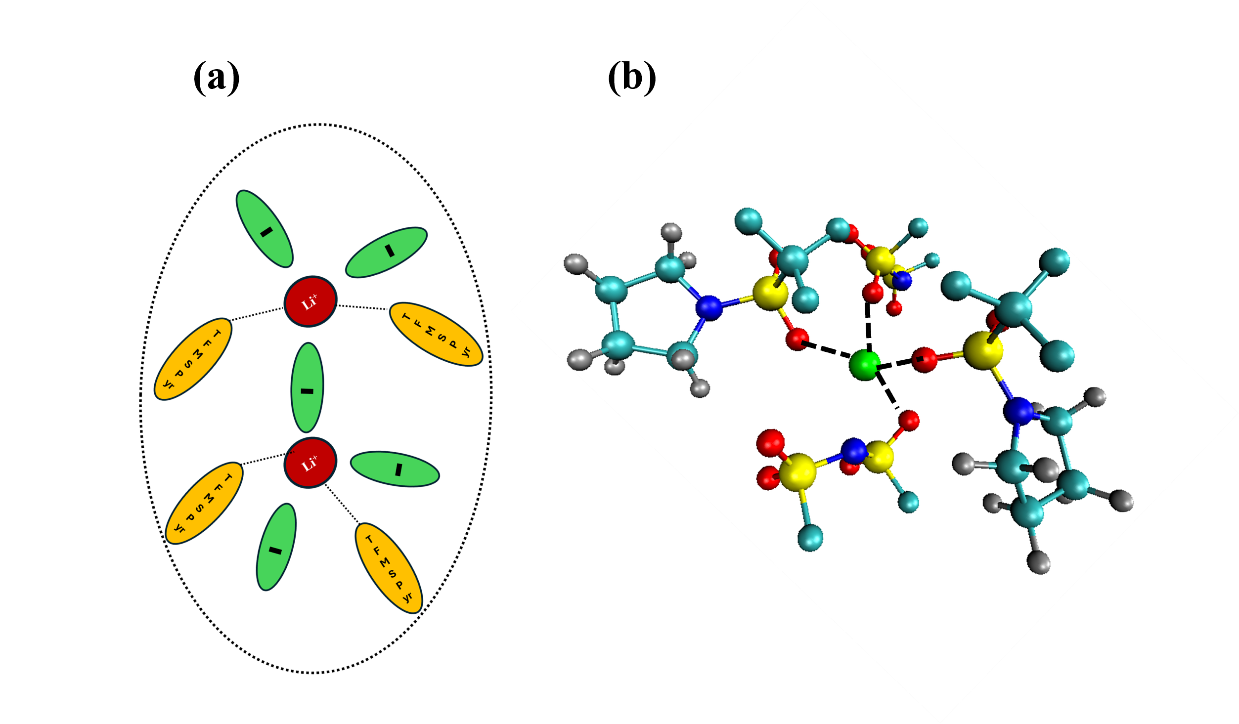 |
| --- |

Figure S5. (a) Schematic representation of weakly-solvating solvation structure of SIPIL10 (1.0 m LiFSI in TFMSPyr), (b) Snapshot of the solvation structure derived from the MD simulation (refer to Figure S1 for the color scheme and representation of the respective atom).

| 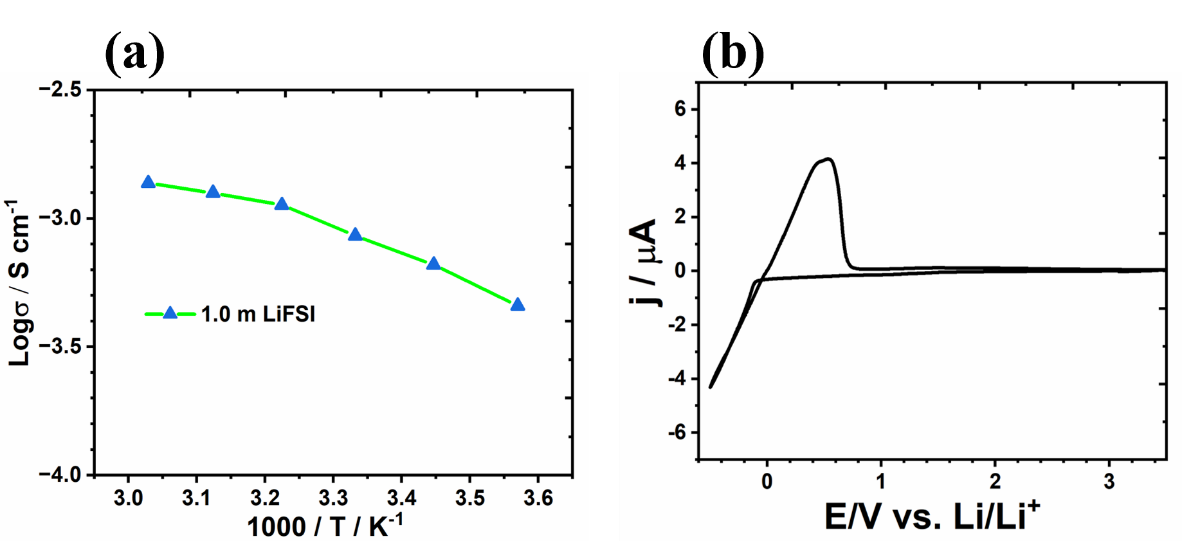 |
| --- |

Figure S6. (a) Temperature dependent ionic conductivity of 1.0 m LiFSI- TFMSPyr from 7 ‒ 57 °C, (b) CV plot of 1.0 m LiFSI- TFMSPyr between - 0.5 V ‒ 3.5 V between Li and stainless-steel electrodes.

Table S4. Temperature dependent ionic conductivity of the 1.0 m LiFSI-TFMSPyr at different temperatures.

| **Temperature / °C** | **Ionic conductivity / mS cm^-1^** |
| --- | --- |
| **57** | **1.37** |
| **47** | **1.25** |
| **37** | **1.12** |
| **27** | **0.85** |
| **17** | **0.66** |
| **7** | **0.45** |

| 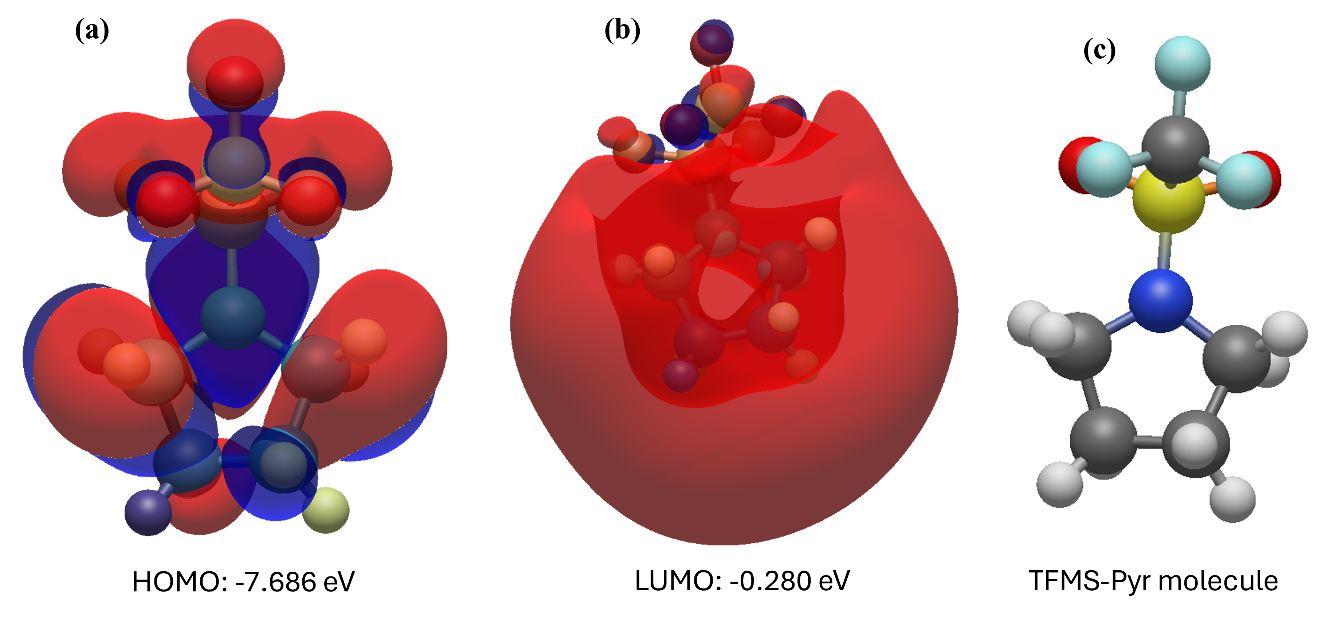 |
| --- |

Figure S7. Density functional theory (DFT) calculation showing (a) Highest occupied molecular orbital (HOMO = -7.686 eV), (b) Lowest unoccupied molecular orbital (LUMO = -0.28), and (c) Lowest energy structure of TFMSPyr, where grey ball represents carbon, white ball represents hydrogen, blue represents nitrogen, yellow represents sulfur, red represents oxygen, and cyan represents fluorine atom.

| 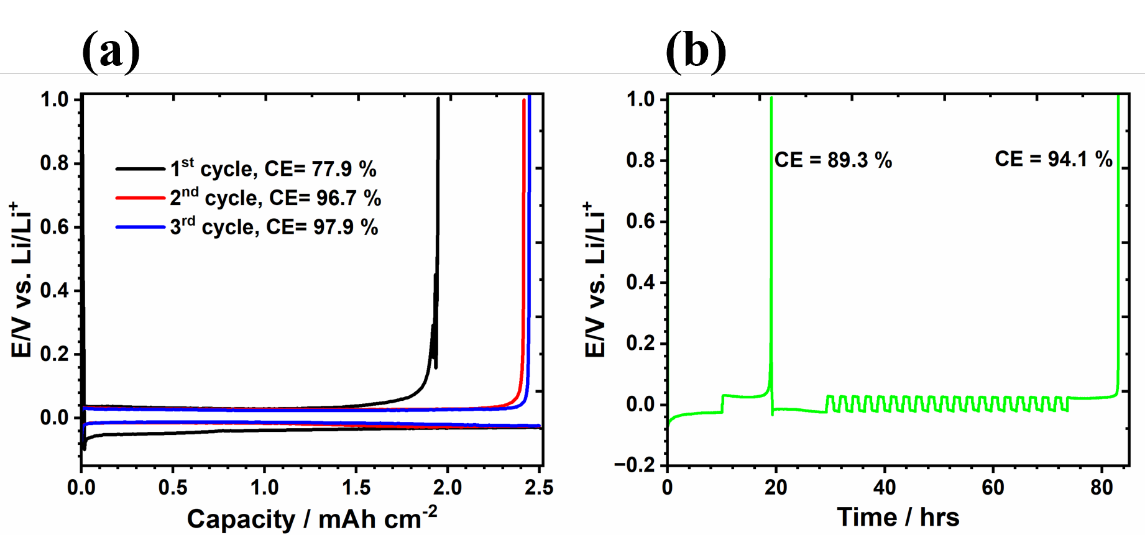 |
| --- |

Figure S8. Electrochemical performance of lithium metal plating / stripping 2.5 mAh cm^-2^ Li at 0.5 mA cm^-2^ on a copper in a two electrode Li|Cu, where Li as counter and reference and Cu as a working electrode. (a) Li plating / stripping voltage profiles, and (b) lithium plating / stripping efficiency measured by Aurbach methods for Li||4 M LiFSI/DME ||Cu cell.


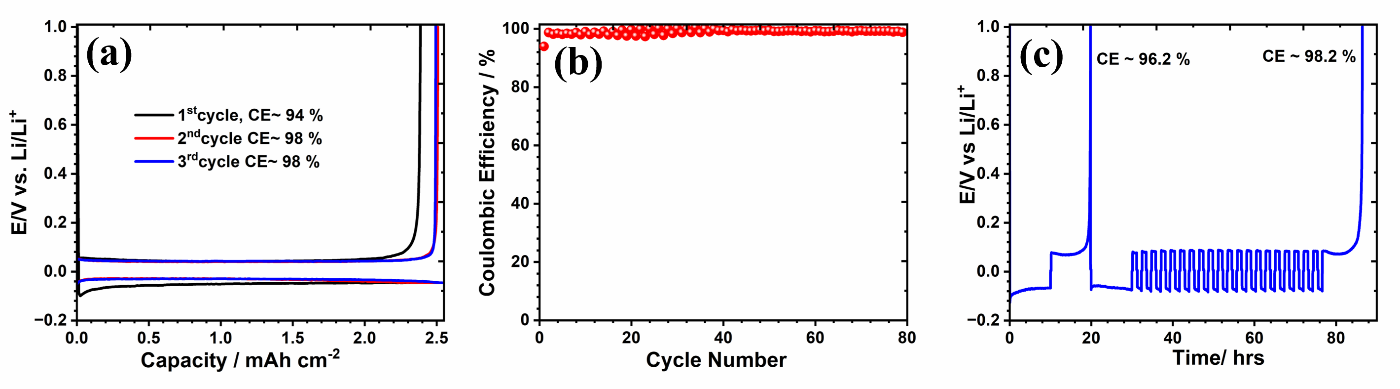


Figure S9. Electrochemical performance of lithium metal plating / stripping 2.5 mAh cm^-2^ Li at 0.5 mA cm^-2^ on a copper in a two electrode Li|Cu, where Li as counter and reference and Cu as a working electrode. (a) Li plating / stripping voltage profiles, (b) coulombic efficiency as a function of cycle number, and (c) lithium plating / stripping efficiency measured by Aurbach methods for Li||SIPIL5||Cu cell.

| 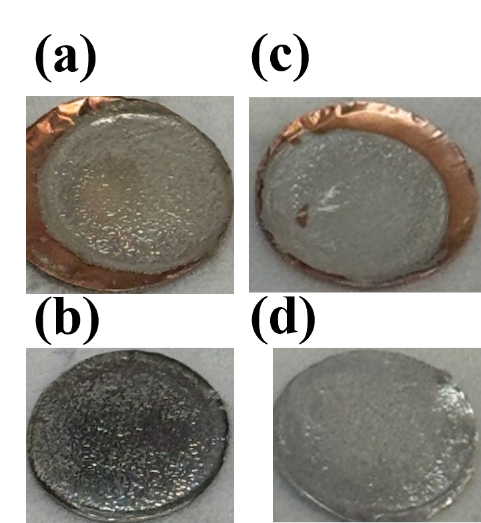 |
| --- |

Figure S10. Digital photographs of (a) plated Li on Copper surface, and (b) Li counter electrode after plating 10 mAh cm^-2^ Li on copper surface at 0.5 mA cm^-2^ with baseline electrolyte for 20 hrs. Digital photographs of (c) plated Li on Copper surface, and (d) Li counter electrode after plating 10 mAh cm^-2^ Li on copper surface at 0.5 mA cm^-2^ with SIPIL10 for 20 hrs.

| 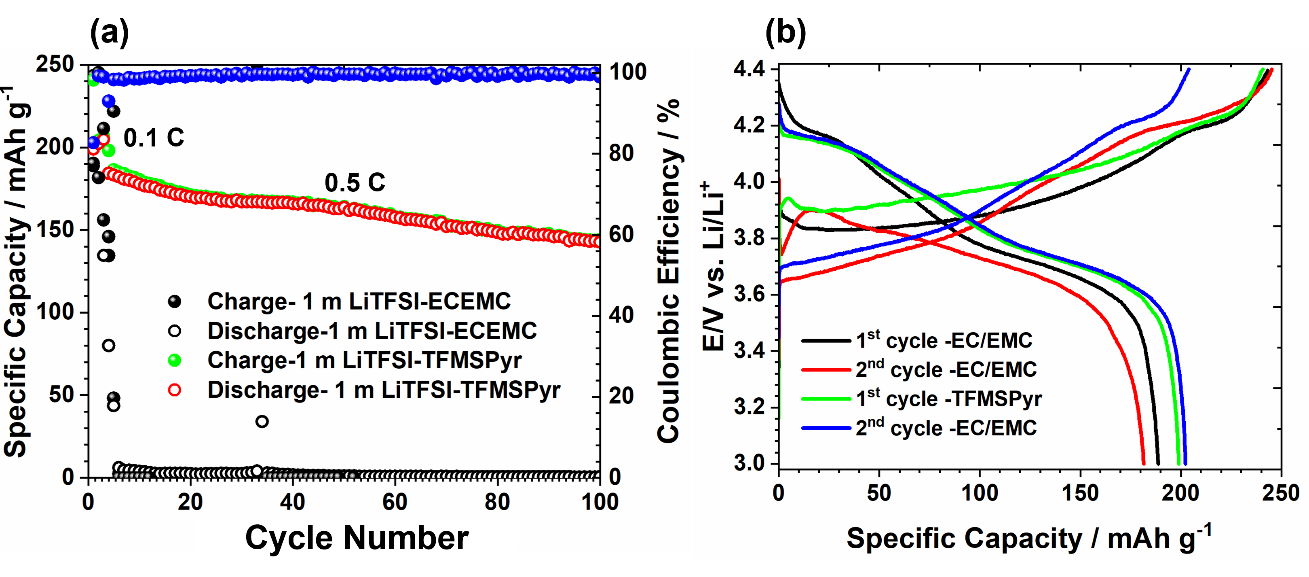 |
| --- |

Figure S11. (a) Specific (charge-discharge) capacities and coulombic efficiencies of Li|| NMC811 cells with 1.0 m LiTFSI in TFMSPyr and EC/EMC (3:7 by volume) electrolyte as a function of cycle number from 3.0 – 4.4 V at 0.1 C for first three cycles, 0.3 C charge and 0.5 C discharge rate for the rest of the cycling period at RT; (b) First two charge-discharge voltage profiles of Li||NMC811 cell cycled at 0.1 C scan rate.

| 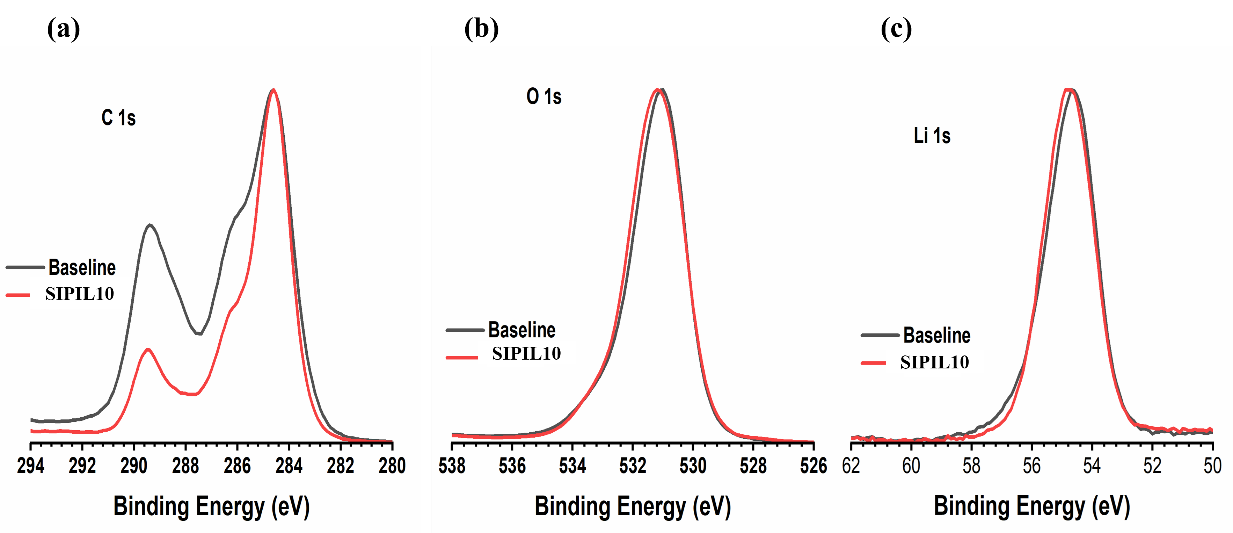 |
| --- |

Figure S12. Core level XPS spectra of SEI of (a) C 1s, (b) O 1s, (c) Li 1s deposited on Li metal from Li||LFP cell for baseline (black) and SIPIL10 (red) electrolyte cycled for three cycles at a scan rate of 0.1 C from 2.8 -3.8 V.

**Preferential FSI decomposition over TFMSPyr study by DFT calculation:**

To understand the relative stabilities of TFMSPyr and FSI anions, we performed a DFT calculation of a reduced radical comprising a lithium ion in the presence of one TFMSPyr molecule and one FSI anion. The Li ion was kept in between the anion and the solvent such that the ion has a maximum probability of binding with the oxygen groups of both TFMSPyr and FSI anion (cf. reactant in the figure below). From simulations of isolated cases of Li-TFMSPyr and Li-FSI systems, the most stable configuration was obtained when the Li-ion coordinated with oxygen atoms. During the optimization, the S-F bond in the FSI molecule, denoted by black oval on the reactant side in the figure below, broke and rearranged with the Li atom to form the LiF radical (cf. product in the figure below). In the meantime, however, none of the bonds in the TFMSPyr molecule broke. Thus, when both the solvent and the anion are near the Li^+^, the radical is formed by spontaneously breaking the S-F bond in FSI^-^ showing qualitatively that TFMSPyr molecules are more stable to decomposition compared with FSI anions. Exact quantification of the transition state pathways requires searching through many different combinations of bond-breakage which is beyond the scope of the current study.


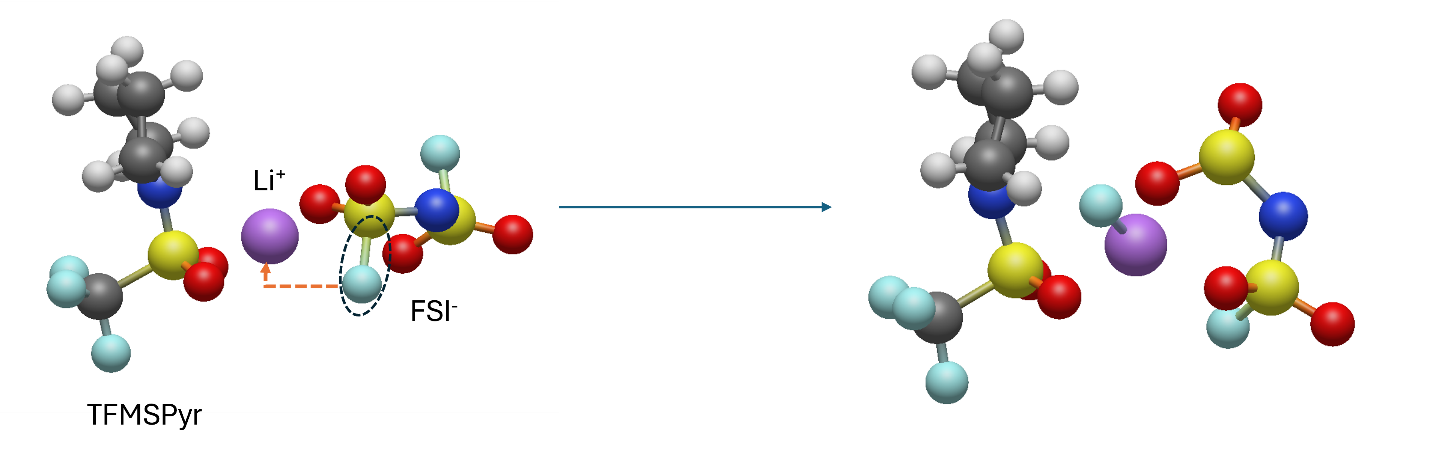


Figure S13. Reaction mechanism depicting the formation of the LiF radical in the presence of TFMSPyr and FSI anions.

**

Figure S14. STEM-EELS spectrum (higher energy range) showing the different elements in the analyzed LFP electrode after 3 charge-discharge cycles in SIPIL10.

Table S5. STEM-EELS of various elemental edges and their respective amounts (at. %) present in the surface of cycled LFP electrode in SIPIL10 for the two energy ranges.

| Element, Shell | Comp. (at. %) | +/- |
| --- | --- | --- |
| *Low loss range* |  |  |
| Fe M | 45 | 5 |
| Li K | 42 | 4 |
| P L | 11.8 | 1.5 |
| C K | 1.9 | 0.2 |
| *High loss range* |  |  |
| C K | 0 | 0.04 |
| N K | 1.26 | 0.1 |
| O K | 81.8 | 1.6 |
| Fe L | 16.9 | 1.6 |

| 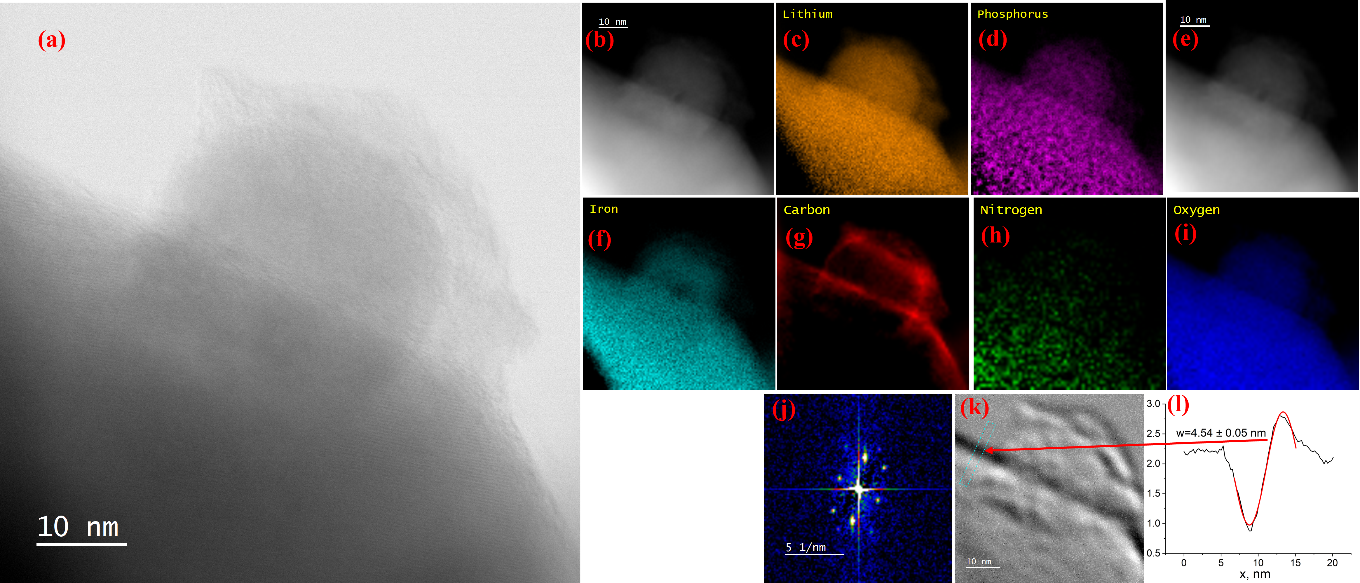 |
| --- |

Figure S15. HR-STEM images of cycled LFP electrode after 3 cycles in baseline electrolyte. (a) ABF image, (b) ADF image acquired simultaneously with EELs data for the lower energy range with (c) Li K and (d) P L edges, (e) ADF image acquired simultaneously with EELs data for the higher energy range with (f) Fe L, (g) C K, (h) N K, and (i) O K edges. CEI thickness measurement from NMF decomposition of HAADF images. (j) Component 2 of a 4-component NMF decomposition of the ABF image, (k) mixing coefficient map corresponding to component 2 with a profile region indicated by a blue rectangle, (l) profile with the corresponding sine fit and width measurement showing the approximate thickness of CEI.

|  |
| --- |

|  |
| --- |

Figure S16. STEM-EELS spectrum (higher energy range) showing the different elements in the analyzed LFP electrode after 3 charge-discharge cycles with baseline electrolytes.

Table S6. STEM-EELS of various elemental edges and their respective amounts (at. %) present in the surface of cycled LFP electrode in baseline electrolyte for the two energy ranges.

| Element, Shell | Comp. (at. %) | +/- |
| --- | --- | --- |
| *Low loss range* |  |  |
| Fe M | 38 | 5 |
| Li K | 44 | 4 |
| P L | 11.5 | 1.4 |
| C K | 6.6 | 0.6 |
| *High loss range* |  |  |
| C K | 14.2 | 0.9 |
| N K | 2.4 | 0.2 |
| O K | 83.5 | 0.9 |

*
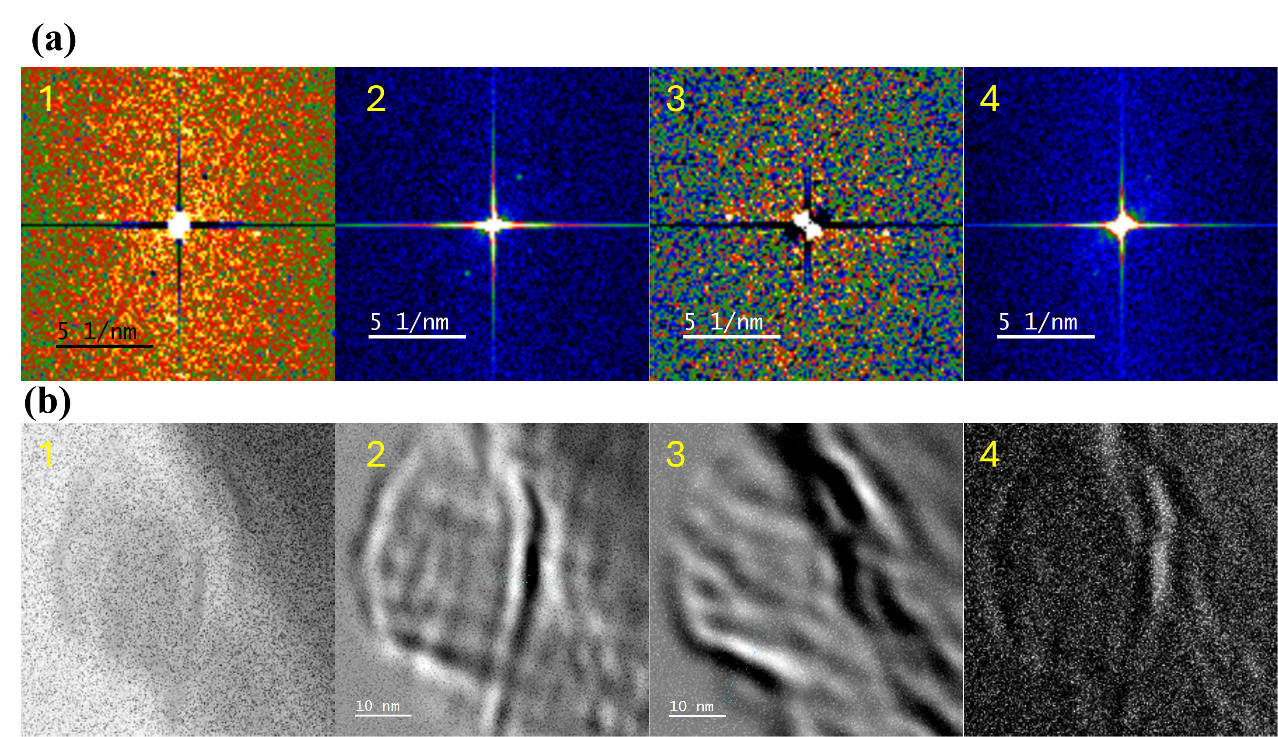
*

Figure S17. Non-negative matrix factorization (NMF) decomposition of the HAADF image in Figure 8 of LFP electrode after 3 charge-discharge cycles with SIPIL10 electrolyte. (a) NMF components numbered; (b) corresponding mixing coefficient maps, numbered. Components 2 and 3 correspond to two different lattice directions; the edge of the respective crystalline regions appear to correspond to the CEI. Components 1 and 4 are amorphous and/or noise components. Since the only crystalline components identified are those of undistorted lattice, it suggests that CEI is not crystalline.

| 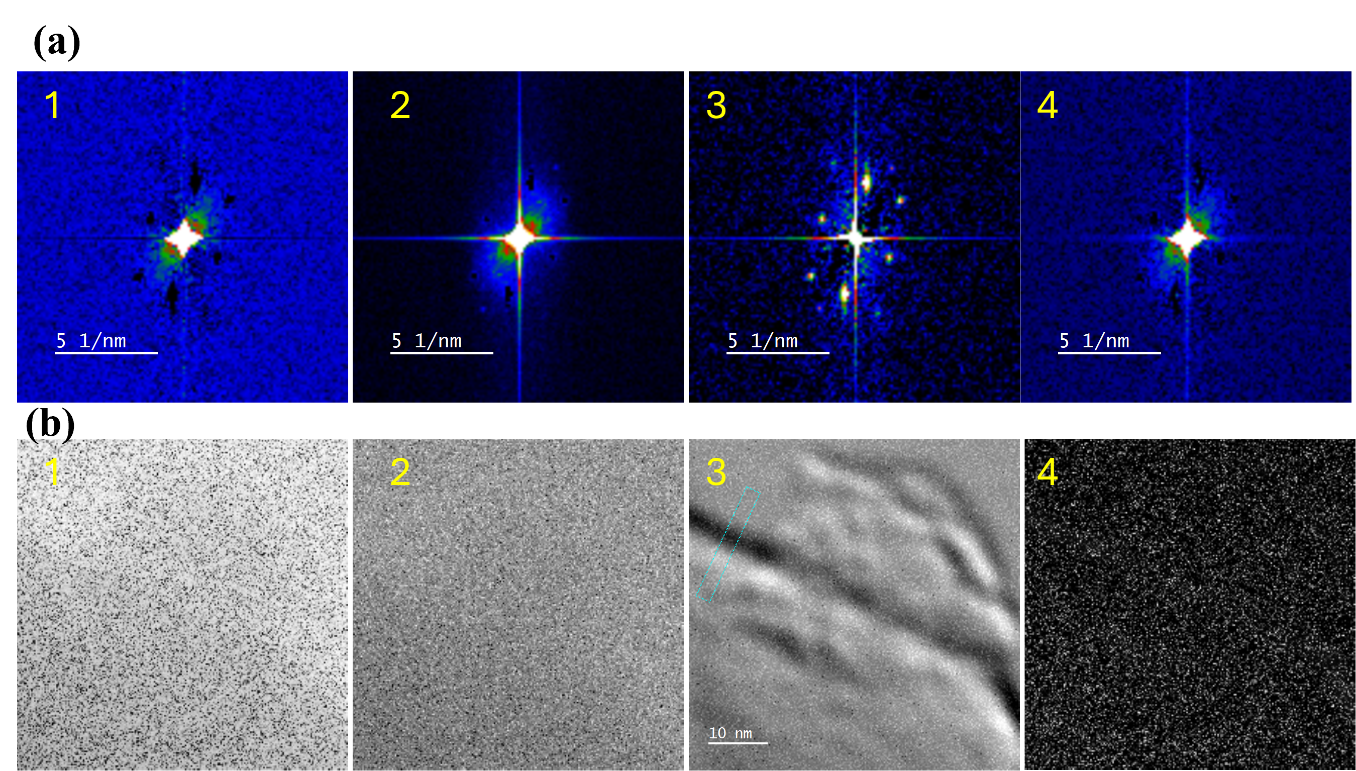 |
| --- |

Figure S18. Non-negative matrix factorization (NMF) decomposition of the ABF image in Figure S15 of LFP electrode after 3 charge-discharge cycles with baseline electrolyte. (a) NMF components numbered; (b) corresponding mixing coefficient maps, numbered. Component 3 corresponds to crystalline region, the edge of which appears to correspond to the CEI, while components 1, 2, and 4 are amorphous and/or noise components. Since the only crystalline component identified is that of undistorted lattice, it suggests that CEI is not crystalline.

Table S7. The electrochemical performance comparison of the TFMSPyr with state-of-art weakly solvating electrolytes reported in the literature

| Weakly solvating Electrolytes | 1^st^ cycle CE of Li\|\|Cu | Av. CE of Li\|\|Cu | CR of Li\|\|LFP |
| --- | --- | --- | --- |
| LHCE (1.2 M LiFSI in DMC-BTFE)[1] |  | 99.3 % (200 cycles) | - |
| 0.3 M LiFSI and 0.2 m LiTFSI in 1,4-Dioxane[2] | - | 99.2 % (100 cycles) | 78.5 % (400 cycles) |
| 1.0 M LiFSI in DEE[3] | 76.0 % | 98.0 % (100 cycles) | 93.5 % (200 cycles) |
| 1.0 M LiFSI in 2-MeTHF[4] | - | 98.8 % | - |
| 1.0 M LiFSI in THP[4] | - | 99.2 % | 93.3 % (200 cycles) |
| 1.0 M LiFSI in 1,4-Dioxane[4] | - | 98.2 % | - |
| 1.0 m LiFSI in TFMSPyr (This work) | 98.8 % | 99.5 % (100 cycles) | 81.6 % (400 cycles) |

LCHE=localized high-concentration electrolytes, DMC=Dimethyl carbonate, DEE= 1,2-diethoxyethane, THP = tetrahydropyran.

Table S8. Cost of the chemicals for the synthesis of TFMSPyr

|  | Chemicals | Purity (%) | Price | Vendor |
| --- | --- | --- | --- | --- |
| Pyrrolidine | Ram materials | ≥ 98.0 % | $ 144.0 / L | Tokyo Chemical Industry |
| Trifluoromethanesulfonyl Chloride | Ram materials | ≥ 99.0 % | $ 9960.0 / kg | Tokyo Chemical Industry |
| Dichloromethane | Solvent | ≥ 90.0 % | $ 62.5 / L | Sigma Aldrich |
| Triethylamine | solvent | Synthesis grade | $ 56.6 /L | Sigma Aldrich |


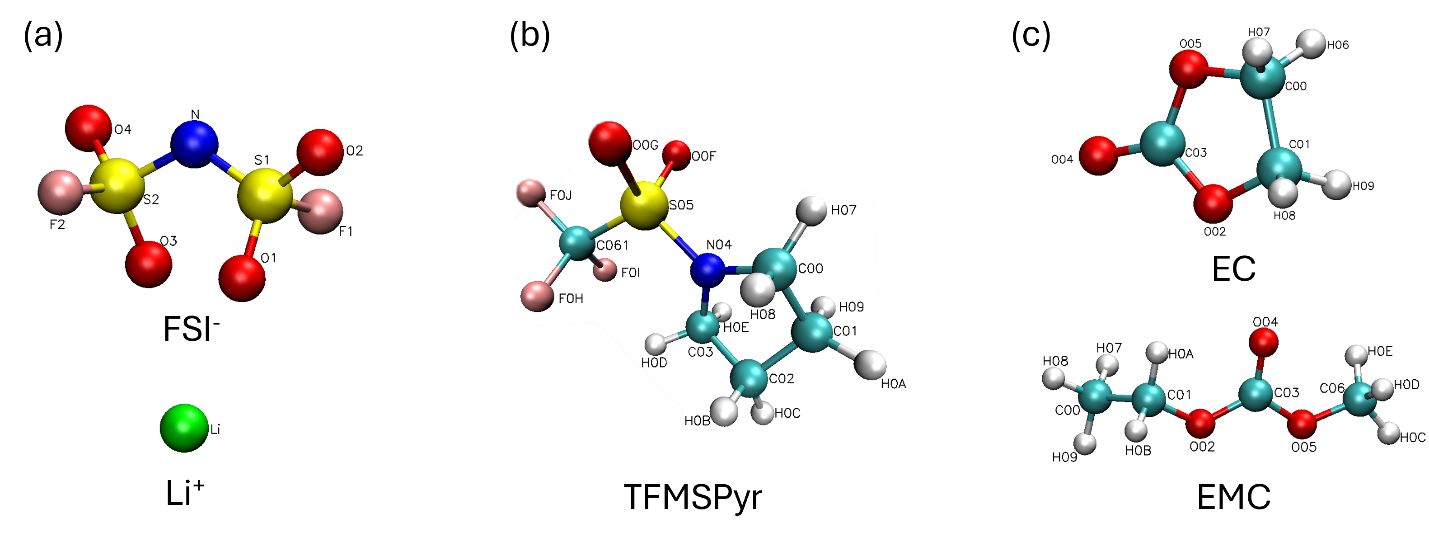


Figure S19: Initial structures and the naming conventions for different molecules used in the simulations: (a) FSI anion (top) and lithium cation (bottom); (b) TFMSPyr solvent and (c) EC (top) and EMC (bottom) solvents.

**Table S9**: Lennard-Jones parameters and charges for different atom-types used in the simulations. The atom names in this table correspond to the naming convention in **Figure S19**.

| Molecule name: Li^+^ | | | | | |
| --- | --- | --- | --- | --- | --- |
| Atom Name | Atom Type | Mass (g/mol) | Charge (q) | $\sigma$ (nm) | $\epsilon$ (kJ/mol) |
| Li | Li | 6.941 | 0.8 | 2.1260E-01 | 7.6480E-02 |
| Molecule name: FSI^-^ | | | | | |
| Atom Name | Atom Type | Mass (g/mol) | Charge (q) | $\sigma$ (nm) | $\epsilon$ (kJ/mol) |
| F1/F2 | FSI | 18.998 | -0.104 | 3.1180E-01 | 2.5540E-01 |
| S1/S2 | SBT | 32.066 | 0.816 | 3.5500E-01 | 1.0460E+00 |
| N | NBT | 14 | -0.528 | 3.2500E-01 | 7.1128E-01 |
| O1/O2/O3/O4 | OBT | 15.999 | -0.424 | 3.1500E-01 | 8.3736E-01 |
| Molecule name: TFMSPyr | | | | | |
| Atom Name | Atom Type | Mass (g/mol) | Charge (q) | $\sigma$ (nm) | $\epsilon$ (kJ/mol) |
| C00 | C800 | 12.011 | 0.073736 | 3.5000E-01 | 2.7614E-01 |
| C01/C02 | C801 | 12.011 | -0.01649 | 3.5000E-01 | 2.7614E-01 |
| C03 | CT803 | 12.011 | 0.073736 | 3.5500E-01 | 2.9288E-01 |
| C06 | C806 | 12.011 | 0.303296 | 3.5000E-01 | 2.7614E-01 |
| N04 | N804 | 14.007 | -0.20176 | 3.2500E-01 | 7.1128E-01 |
| O0F/O0G | O815 | 15.999 | -0.37929 | 2.9600E-01 | 7.1128E-01 |
| S05 | S805 | 32.06 | 0.695376 | 3.5500E-01 | 1.0460E+00 |
| F0H/F0I/F0J | F817 | 18.9984 | -0.11682 | 2.9000E-01 | 2.5104E-01 |
| H07/H08 | H807 | 1.008 | 0.027512 | 2.5000E-01 | 1.2552E-01 |
| H09/H0A | H809 | 1.008 | 0.027328 | 2.5000E-01 | 1.2552E-01 |
| H0B/H0C | H811 | 1.008 | 0.016912 | 2.5000E-01 | 1.2552E-01 |
| H0D/H0E | H813 | 1.008 | 0.027056 | 2.5000E-01 | 1.2552E-01 |
| Molecule name: EC | | | | | |
| Atom Name | Atom Type | Mass (g/mol) | Charge (q) | $\sigma$ (nm) | $\epsilon$ (kJ/mol) |
| C00 | C800 | 12.011 | 0.13864 | 3.5000E-01 | 2.7614E-01 |
| C01 | C801 | 12.011 | 0.12617 | 3.5000E-01 | 2.7614E-01 |
| O02 | O802 | 15.999 | -0.33706 | 2.9000E-01 | 5.8576E-01 |
| C03 | C803 | 12.011 | 0.74599 | 3.5500E-01 | 2.9288E-01 |
| O04 | O804 | 15.999 | -0.4725 | 2.9600E-01 | 8.7864E-01 |
| O05 | O805 | 15.999 | -0.33904 | 2.9000E-01 | 5.8576E-01 |
| H06 | H806 | 1.008 | 0.03294 | 2.5000E-01 | 1.2600E-01 |
| H07 | H807 | 1.008 | 0.03294 | 2.5000E-01 | 1.2552E-01 |
| H08/H09 | H808 | 1.008 | 0.03596 | 2.5000E-01 | 1.2552E-01 |
| Molecule name: EMC | | | | | |
| Atom Name | Atom Type | Mass (g/mol) | Charge (q) | $\sigma$ (nm) | $\epsilon$ (kJ/mol) |
| C00 | C800 | 12.011 | -0.21525 | 3.5000E-01 | 2.7614E-01 |
| C01 | C801 | 12.011 | 0.364093 | 3.5000E-01 | 2.7614E-01 |
| O02 | O802 | 15.999 | -0.43835 | 2.9000E-01 | 5.8576E-01 |
| C03 | C803 | 12.011 | 0.799609 | 3.5500E-01 | 2.9288E-01 |
| O04 | O804 | 15.999 | -0.47795 | 2.9600E-01 | 8.7864E-01 |
| O05 | O805 | 15.999 | -0.34816 | 2.9000E-01 | 5.8576E-01 |
| C06 | C806 | 12.011 | 0.068504 | 3.5000E-01 | 2.7614E-01 |
| H07/H08/H09 | H807 | 1.008 | 0.056789 | 2.5000E-01 | 1.2552E-01 |
| H0A/H0B | H810 | 1.008 | -0.02507 | 2.5000E-01 | 1.2552E-01 |
| H0C/H0D/H0E | H812 | 1.008 | 0.042424 | 2.5000E-01 | 1.2552E-01 |

**Table S10**: Total number of Li-FSI and solvent molecules and the initial box length used for a concentration of 1 m in simulations.

| For Li-TFMSPyr system (Solvent: TFMSPyr) | | | | | | |
| --- | --- | --- | --- | --- | --- | --- |
| Li atoms | FSI molecules | TFMSPyr molecules | | Total # of atoms | | Initial box length (Å) |
| 67 | 67 | 300 | | 6670 | | 51 |
| For Li-EC/EMC system (Solvent: EC:EMC in 3:7 weight ratio) | | | | | | |
| Li atoms | FSI molecules | EC molecules | EMC molecules | | Total # of atoms | Initial box length (Å) |
| 59 | 59 | 200 | 395 | | 8515 | 49 |

**References**

[1] S. Chen, J. Zheng, D. Mei, K. S. Han, M. H. Engelhard, W. Zhao, W. Xu, J. Liu, J.-G. Zhang, *Advanced Materials* **2018**, *30* (21), 1706102, <https://doi.org/https://doi.org/10.1002/adma.201706102>.

[2] T. D. Pham, A. Bin Faheem, H. D. Nguyen, H. M. Oh, K.-K. Lee, *Journal of Materials Chemistry A* **2022**, *10* (22), 12035, <https://doi.org/10.1039/D2TA02743G>.

[3] T. D. Pham, K.-K. Lee, *Small* **2021**, *17* (20), 2100133, <https://doi.org/https://doi.org/10.1002/smll.202100133>.

[4] Y. Liao, M. Zhou, L. Yuan, K. Huang, D. Wang, Y. Han, J. Meng, Y. Zhang, Z. Li, Y. Huang, *Advanced Energy Materials* **2023**, *13* (32), 2301477, <https://doi.org/https://doi.org/10.1002/aenm.202301477>.
